# Electro‐ and Photo‐ Dual Responsive Chromatic Devices for High‐Contrast Dimmers

**DOI:** 10.1002/adma.202410703

**Published:** 2024-12-30

**Authors:** Bin Wang, Pengcheng Liu, Feifei Zhao, Bingkun Huang, Wu Zhang, Abdulhakem Y. Elezzabi, Linhua Liu, William W. Yu, Haizeng Li

**Affiliations:** ^1^ Institute of Frontier & Interdisciplinary Science Shandong University Qingdao 266237 China; ^2^ School of Energy and Power Engineering Shandong University Jinan Shandong 250061 China; ^3^ School of Chemistry & Chemical Engineering Shandong University Jinan 250100 China; ^4^ Ultrafast Optics and Nanophotonics Laboratory Department of Electrical and Computer Engineering University of Alberta Edmonton AB T6G 2V4 Canada

**Keywords:** AR glass, dynamic windows, electrochromic devices, photochromic devices, VR glass

## Abstract

Electrochromism stands out as a highly promising technology for applications including variable optical attenuators, optical switches, transparent displays, and dynamic windows. The pursuit of high‐contrast tunability in electrochromic devices remains a challenging goal. Here, the first photochromic hydrogel electrolyte is reported for electro‐ and photo‐dual responsive chromatic devices that yield a high transmittance contrast at 633 nm (ΔT = 83.1%), along with a tinted transmittance below 1.5%. Such high‐contrast devices not only hold great promise for dynamic windows but also enable seamless transitions between transparent augmented reality (AR) glass and opaque virtual reality (VR) glass. These findings introduce an innovative strategy for the design of high‐contrast dimmers, opening new avenues for the development of chromatic devices.

## Graphical abstract

A highly transparent photochromic hydrogel electrolyte is developed, which not only serves as an electrolyte for zinc anode‐based electrochromic devices but also provides a photochromic effect to reduce tinted transmittance. These dual‐responsive dimmers address concerns about low transmittance contrast in electrochromic devices, representing a significant step forward for high‐contrast dimming in dynamic windows and AR glasses.

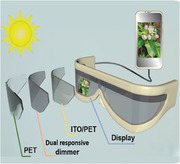

## 1 Introduction

Electrochromic dimmers hold great promise for dynamic windows in green buildings, as well as for lenses in augmented reality (AR) glasses, owing to their ability to control light transmittance.^[^ ^1^ ^]^ Achieving a high transmittance contrast at the device level remains a challenging goal due to the relatively high tinted transmittance and the low bleached transmittance. The utilization of dual electrochromic electrodes in an emerging zinc anode‐based electrochromic device has significantly reduced tinted transmittance.^[^ ^2^ ^]^ However, this approach also introduces transmittance loss in bleached state due to the presence of two electrochromic electrodes.^[^ ^2^ ^]^

Since the dual electrochromic electrode strategy yields minimal improvement in the optical contrast of a device, for example, the optical contrasts of KVO‐Zn‐KVO display,^[^ ^3^ ^]^ SVO‐Zn‐SVO display^[^ ^4^ ^]^ and CWO‐Zn‐CWO window^[^ ^5^ ^]^ are 18%, 20%, and 65% respectively, exploring novel approaches warrants further investigation. The introduction of thermochromic gel electrolytes into electrochromic devices has significantly enhanced their opaque properties, attributed to the light scattering of the polymer chains.^[^ ^6^ ^]^ Unfortunately, such light scattering would significantly impact the field of view,^[^ ^7^ ^]^ thus reducing the comfortable experience of the human‐machine interface when utilizing the devices in AR glasses. For example, the thermochromic gel electrolytes become opaque at high temperatures, which greatly limits the use of AR glasses outdoors in the summer. Additionally, although it is reported that reversible metal electrodeposition combined with ion insertion electrochromism delivers low tinted transmittance,^[^ ^8^ ^]^ the challenge of reduced bleached transmittance remains significant.

The transmittance loss of the electrochromic devices in bleached state is mainly attributed to the indium tin oxide (ITO) layers. Reducing the use of ITO layers could maximize the bleached transmittance of the electrochromic devices and also significantly lower the production cost of the devices.^[^ ^9^ ^]^ The newly developed zinc anode‐based electrochromic device presents an opportunity to eliminate the use of the counter electrode (e.g., NiO‐coated ITO glass).^[^ ^10^ ^]^ By tailoring the zinc anode to the frame structure, the bleached transmittance of the device could be improved. Besides the transmittance loss from electrodes, the electrolyte transmittance loss also affects the devices’ transmittance contrast. Among current electrolyte options, hydrogel electrolytes are promising candidates for next‐generation electrochromic devices due to their excellent ionic conductivity and high transparency.^[^ ^11^ ^]^ Therefore, incorporating reversible color change capability into hydrogel electrolytes is considered a versatile method to enhance the transmittance contrast of electrochromic devices.

Herein, the photochromic hydrogel electrolyte for electrochromic devices is designed for the first time. In comparison to previous reports, the approach of incorporating photochromic functionality into electrochromic devices through gel electrolytes represents a novel attempt (Table S1, Supporting Information). Such a photochromic hydrogel electrolyte in an electrochromic device, composed of a polyacrylamide (PAAm) matrix, N,N‐methylene bisacrylamide (MBAA) crosslinker and well‐designed ethylene glycol (EG)‐capped WO_3_ nanodots,^[^ ^12^ ^]^ enables low tinted transmittance due to the photochromic function without sacrificing bleached transmittance. Additionally, the photochromic electrolyte delivers a high ionic conductivity (1.08 S m^−1^ at 20 °C) compared to state‐of‐the‐art electrolytes.^[^ ^13^ ^]^ We unveil that the incorporation of EG‐capped WO_3_ colloid significantly enhances the mechanical strength of the hydrogel electrolyte (e.g., stretchability, self‐healing capability), along with exhibiting excellent reversible sunlight‐driven photochromism (ΔT = 90.2% at 633 nm). These superior mechanical properties and photochromic capabilities make the photochromic hydrogel electrolyte a promising candidate for electrochromic devices, enabling the construction of electro‐ and photo‐dual‐responsive chromatic devices. As a proof of concept, the Zn‐WO_3_ electrochromic device, equipped with the photochromic hydrogel electrolyte, achieves a contrast of 83.1% at 633 nm by overlaying the electrochromic and photochromic color effects. Furthermore, the Zn‐WO_3_ dimmer exhibits high transparency in the bleached state (85.3% at 633 nm) and shows rapid switching times at the 5 by 5 cm device scale (t_c_ = 8.1 s for coloration and t_b_ = 5.8 s for bleaching). These results reveal that the Zn‐WO_3_ dimmer has excellent overall performance in comparison to previously reported devices (Table S2, Supporting Information). This dual‐responsive Zn‐WO_3_ dimmer can also be applied to flexible substrates, leveraging the excellent mechanical strength of the photochromic hydrogel electrolyte. These flexible devices have potential applications as dimming lenses in AR glasses, facilitating the adjustment of brightness levels between virtual images and physical objects. Commercial AR glasses, integrated with the flexible Zn‐WO_3_ dual‐responsive dimmer, can offer four dimming levels, enabling seamless transitions between transparent AR and opaque virtual reality (VR) modes.

## 2 Results

### 2.1 Fabrication and Characterization of EG/PAAm Photochromic Hydrogel Electrolyte

**Figure 1:**
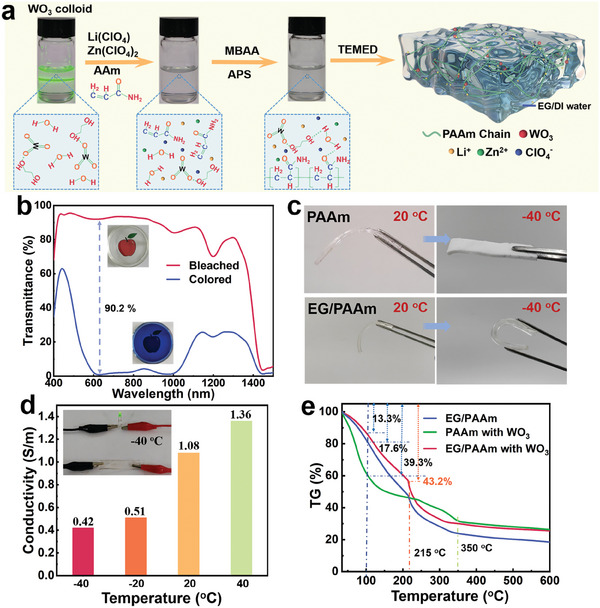
Preparation and characterization of the EG/PAAm photochromic hydrogel. a) A schematic illustration outlines the preparation procedure of the EG/PAAm photochromic hydrogel. b) The optical transmittance spectra of the EG/PAAm hydrogel electrolyte in different color states. Inset: corresponding digital photographs of the hydrogel electrolyte. c) Digital photographs of morphological changes of EG/PAAm photochromic hydrogel and pure PAAm hydrogel at 20 and −40 °C, respectively. d) The conductivity of the EG/PAAm hydrogel at different temperatures. Inset: LED illumination by the EG/PAAm photochromic hydrogel as a conductor at – 40 °C. e) Thermogravimetric Analysis (TGA) curves represent the thermal stability of the EG/PAAm hydrogel without WO_3_, the pure PAAm hydrogel containing WO_3_, and the EG/PAAm hydrogel containing WO_3_.

The photochromic hydrogel electrolyte was fabricated using a modified and environmentally friendly approach, which aligns with the well‐established design principles mentioned in the previous report.^[^ ^14^ ^]^ In line with the detailed fabrication approach depicted in **Figure** **1**a, a highly transparent colloid of EG‐capped WO_3_ nanodots^[^ ^12^ ^]^ served as the starting precursor to fabricate the photochromic hydrogel electrolyte. The EG‐capped WO_3_ nanodots, with diameters ranging from 2 to 5 nm (Figure S1, Supporting Information), functioned as the photochromic component. We employed Fourier transform infrared spectroscopy (FTIR) to investigate the capping effectiveness of EG. As illustrated in Figure S2 (Supporting Information), the spectrum of the EG‐capped WO_3_ colloid includes all characteristic peaks of EG, along with a faint peak at 1658 cm^−1^, associated with the −C═O bond. The presence of this C═O bond suggests that a small portion of the hydroxyl groups in EG have undergone dehydrogenation, forming carbonyl groups. This observation reveals that the oxygen atoms in WO_3_ form hydrogen bonds with hydroxyl groups from EG, thereby providing additional electrons to WO_3_.^[^ ^12^ ^]^ Consequently, this result confirms the bond between EG and WO_3_, which enables EG to cap around WO_3_. To enhance the ionic conductivity of the electrolyte, lithium perchlorate (Li(ClO_4_)) and zinc perchlorate (Zn(ClO_4_)_2_) were incorporated into the colloid to form a clear solution. The choice of cations for the electrolytes included Li^+^ and Zn^2+^, as they are commonly used for coloration in electrochromic materials^[^ ^15^ ^]^ and for charge balance in the zinc anode,^[^ ^2^, ^16^ ^]^ respectively. Perchlorates (ClO_4_^−^) are interesting polyatomic anions used as they allow for the design of robust dynamic windows with long shelf lives,^[^ ^17^ ^]^ as well as high reversible zinc stripping/platting.^[^ ^18^ ^]^

With the excellent photochromic component and proper conducting cations and anions successfully determined, we next investigate the appropriate hydrogel polymer matrix for constructing photochromic hydrogel electrolytes. It is reported that the PAAm matrix delivers excellent mechanical properties and high ionic conductivity,^[^ ^19^ ^]^ as well as ultrahigh transparency,^[^ ^20^ ^]^ making it suitable for constructing electro‐ and photo‐dual responsive chromatic devices. Then, acrylamide (AAm) monomer, N, N′‐methylene MBAA crosslinker, ammonium persulfate (APS, (NH_4_)_2_S_2_O_8_) initiator, and tetramethyl‐ethylenediamine (TEMED) catalyst are subsequently dissolved into the clear colloid of EG‐capped WO_3_ nanodots containing Li(ClO_4_) and Zn(ClO_4_)_2_. Afterward, the above precursor was poured into a plastic mold and thermally heated at 40 °C for 20 min to finalize the hydrogel fabrication process, forming the photochromic hydrogel electrolyte (i.e., PAAm hydrogel).

The Zn^2+^/ Li^+^ PAAm hydrogel exhibits significantly higher transparency (>94% within the visible light region, Figure 1b) compared to that of our recently reported polyvinyl alcohol (PVA)‐based photochromic hydrogels (< 85% within the visible light region),^[^ ^12^ ^]^ representing nearly a 10% enhancement in transmittance. Furthermore, the highly transparent PAAm hydrogel exhibits an obvious color change when exposed to sunlight, delivering a contrast of 90.2% at 633 nm. More importantly, the Zn^2+^/ Li^+^ PAAm hydrogel exhibits a rapid light response, achieving noticeable light modulation within 30 s, as illustrated by the transmission spectra for varying durations of light exposure presented in Figure S3 (Supporting Information). The significant improvement in the photochromic performance of the PAAm hydrogel is attributed to its outstanding water retention capacity compared to that of the PVA hydrogel^[^ ^11a^ ^]^ and the choice of proper cations. The Li^+^, having a smaller radius (0.076 nm, smaller than hydrogel ions), may contribute to the photochromic effect. As a result, the PAAm hydrogel electrolyte containing pure Zn^2+^ exhibits an inferior photochromic effect, with a transmittance contrast of △T = 65.1% at 633 nm (Figure S4a, Supporting Information). Such a value is much lower than that of the Zn^2+^/Li^+^ PAAm hydrogel electrolyte (△T = 90.2% at 633 nm of, Figure 1b). Additionally, while Al^3+^ exhibits higher electrochemical activity toward WO_3_,^[^ ^21^ ^]^ its inclusion in the hydrogel reduces the photochromic effect and slows the hydrogel formation, requiring 1 h for polymerization. This is attributed to the hydrolysis of Al^3+^, which reduces the number of free water molecules that bridge the EG‐capped WO_3_ and PAAm chains during the polymerization process.^[^ ^22^ ^]^ As shown in Figure S4b (Supporting Information), the Zn^2+^/Al^3+^ PAAm hydrogel electrolyte also exhibits an inferior photochromic transmittance contrast (△T = 78.1% at 633 nm) compared to that of the Zn^2+^/ Li^+^ PAAm hydrogel electrolyte (△T = 90.2% at 633 nm, Figure 1b). However, the complete fading of the Zn^2+^/Li^+^ PAAm hydrogel requires 5 h (Figure S5, Supporting Information), which poses a challenge for practical applications. Consequently, future work should focus on reducing the fading time of the hydrogel.

In addition, utilizing the colloid of EG‐capped WO_3_ nanodots, consisting of EG and DI water, brings additional anti‐freezing and self‐healing functionalities. Figure 1c compares the morphological changes of the EG/PAAm photochromic hydrogel and pure PAAm hydrogel. The EG/PAAm photochromic hydrogel retains flexibility at –40 °C, while the pure PAAm hydrogel becomes frozen. To elucidate the mechanism underlying the low‐temperature flexibility of the EG/PAAm hydrogel, we analyzed its structural characteristics alongside those of the pure PAAm hydrogel using FTIR (Figure S6, Supporting Information). The EG/PAAm hydrogel showed obvious PAAm characteristic peaks,^[^ ^23^ ^]^ confirming that polyacrylamide remains as the structural framework, which ensures high transmittance. In addition, characteristic peaks of EG were also observed in the EG/PAAm hydrogel. Notably, the hydroxyl stretching vibration peak shifted from 3227 cm^−1^ to a lower frequency of 3195 cm^−1^, indicating that EG enhanced the hydrogen bond interaction with the polymer network.^[^ ^24^ ^]^ These hydrogen bonds can diminish the interactions between water molecules, and lower the crystallization point of the hydrogel. Moreover, these sacrificial bonds can effectively dissipate external energy during deformation.^[^ ^22^, ^25^ ^]^ The combination of covalent cross‐linking bonds and physical hydrogen bonds endows the hydrogels' excellent low‐temperature flexibility. As a result, the pure PAAm hydrogel can be easily freeze‐dried to observe the porous cross‐sectional scanning electron microscopy (SEM) image (Figure S7a, Supporting Information). In contrast, the EG/PAAm hydrogel exhibits a flat morphology (Figure S7b, Supporting Information). Based on the excellent anti‐freezing capability, the EG/PAAm photochromic hydrogel at −40 °C can also light up the light‐emitting diode (LED) (inset in Figure. 1d), demonstrating its anti‐freezing conductivity. We then investigated the ionic conductivity of the EG/PAAm photochromic hydrogel within the temperature range of 40 to – 40 °C (Figure 1d; Figure S8, Supporting Information). The EG/PAAm photochromic hydrogel provides ionic conductivities as high as 1.08 S m^−1^ at 20 °C while remaining at 0.42 S m^−1^ at – 40 °C. These values are all superior to those of the state‐of‐the‐art electrolytes,^[^ ^13^, ^26^ ^]^ making the EG/PAAm photochromic hydrogel suitable for dynamic windows used in high latitudes.Similar research had been reported previously by J. Li and coauthors, where the OHGel‐8(PAMPS(poly(2‐acrylamido‐2‐methylpropane sulfonic acid))/PAAm hydrogel contain LiCl of 8 mol L^−1^) exhibits high mechanical properties, low‐temperature flexibility (−80 to 120 °C), and excellent conductivity after dehydration treatment (5.6 S m^−1^ at room temperature).^[^ ^27^ ^]^ However, the flexibility of the OHGel‐8 is due to the dual network structure of PAMPS and PAAm, while EG serves as an additive to prevent freezing.^[^ ^27^ ^]^ In this work, the incorporation of EG in EG/PAAm hydrogel not only enhanced the photochromic performance of WO_3_ nanodots but also maintained the flexibility of the PAAm single network structure.

As depicted in Figure 1e, the volatilization temperature of EG within the EG/PAAm hydrogel is 215 °C. Before reaching this temperature, the reduction in mass of the EG/PAAm hydrogel is primarily attributed to water evaporation. Accordingly, the water content of the EG/PAAm hydrogel is estimated to be 43.2%. Furthermore, the EG/PAAm photochromic hydrogel only loses 13.3% of free water at 100 °C, which is superior to the water loss of 39.3% in PAAm hydrogel (Figure 1e), indicating its good water retention at high temperatures. Thus, the EG/PAAm photochromic hydrogel can be employed in both winter and summer. Although the EG/PAAm photochromic hydrogel possesses good water retention at high temperatures, water loss at room temperature can also affect the ECD performance. Therefore, we compare the mass changes of the two hydrogels at room temperature within 17 days. As shown in Figure S9 (Supporting Information), the specific gravity of water loss in EG/PAAm hydrogel (11.8%) is significantly lower than that observed in pure PAAm hydrogel (65.3%). It indicates that the EG/PAAm hydrogel has excellent water retention properties at room temperature.

### 2.2 Mechanical Properties of EG/PAAm Photochromic Hydrogel Electrolyte

**Figure 2:**
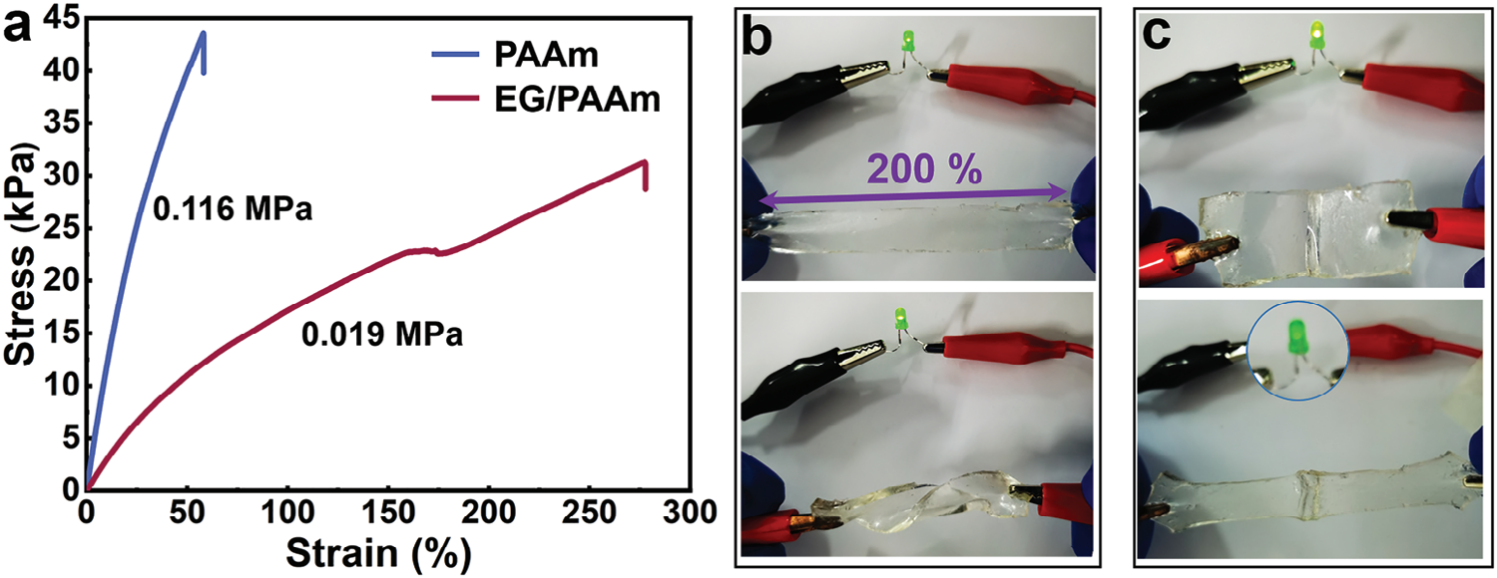
Characterization of physical properties of EG/PAAm hydrogel electrolyte. a) Stress–strain curves of the EG/PAAm hydrogel electrolyte and pure PAAm hydrogel electrolyte. b) Digital photographs of the EG/PAAm hydrogel electrolyte showing its stretchability and twistability. c) Digital photographs illustrating the self‐healing capability of the EG/PAAm hydrogel.

Apart from its excellent photochromic effect, anti‐freezing capability, and ionic conductivity, the EG/PAAm hydrogel also demonstrates good mechanical properties, making it suitable for constructing flexible electro‐ and photo‐dual responsive chromatic devices. To quantitatively investigate the mechanical properties of the hydrogels, tensile stress‐strain measurements are conducted at room temperature. **Figure** **2**a reveals that the EG/PAAm hydrogel delivers the mechanical performance with a maximum tensile strength of 31.2 kPa, 277% elongation at break, and 0.019 MPa Young's modulus (red line in Figure 2a) at a stretching rate of 1 mm min^−1^. The tensile performance of EG/PAAm hydrogel is superior to that of the pure PAAm hydrogel (fracture strain is 58% in Figure 2a). This enhancement of the mechanical performance is attributed to the inclusion of EG, which generates hydrogen‐bond interactions between the organic molecules of EG and the polymer matrix.^[^ ^12^, ^28^ ^]^ Additionally, we investigated the changes in transmittance and tensile strength of the EG/PAAm hydrogel under different temperature conditions (−20, −40, 50, and 80 °C). As shown in Figure S10a (Supporting Information), even at 80 °C, the transmittance of the EG/PAAm hydrogel at 633 nm remained above 60%, while at −40 °C, the transmittance reached 80%. We adjusted the sample dimensions to meet the requirements of in‐situ temperature tensile testing. At 80 °C, the EG/PAAm hydrogel exhibited a tensile elongation at break of only 51.5% and a tensile strength of 8.2 kPa; however, the EG/PAAm hydrogel maintained excellent mechanical properties even at −40 °C. Therefore, the enhancement of the EG/PAAm hydrogel's mechanical properties under extreme conditions should be the focus of future research endeavors. Figure 2b shows the stretchability and twistability of the EG/PAAm photochromic hydrogel, demonstrating its capability to maintain illumination of a connected LED without noticeable alteration in light intensity. In contrast, the pure PAAm hydrogel lacks such stretchability (Figure S11, Supporting Information). Additionally, the hydrogen bonds between EG and water molecules facilitate reversible dynamic interactions,^[^ ^29^ ^]^ which endow the self‐healing capability of the EG/PAAm hydrogel. The schematic diagram in Figure S12 (Supporting Information) shows the self‐healing performance of the EG/PAAm hydrogel and its remarkable elasticity after self‐healing. As shown in Figure S12b (Supporting Information), the severed EG/PAAm hydrogel remains functional as a conductor for illuminating the LED when rejoined for 10 min (Figure 2c). Moreover, the EG/PAAm hydrogel can regain its stretchability after a 4 h healing period (Figure 2c; Figure S12c, Supporting Information).

### 2.3 Electrochromic Performance of WO_3_ Cathode

**Figure 3:**
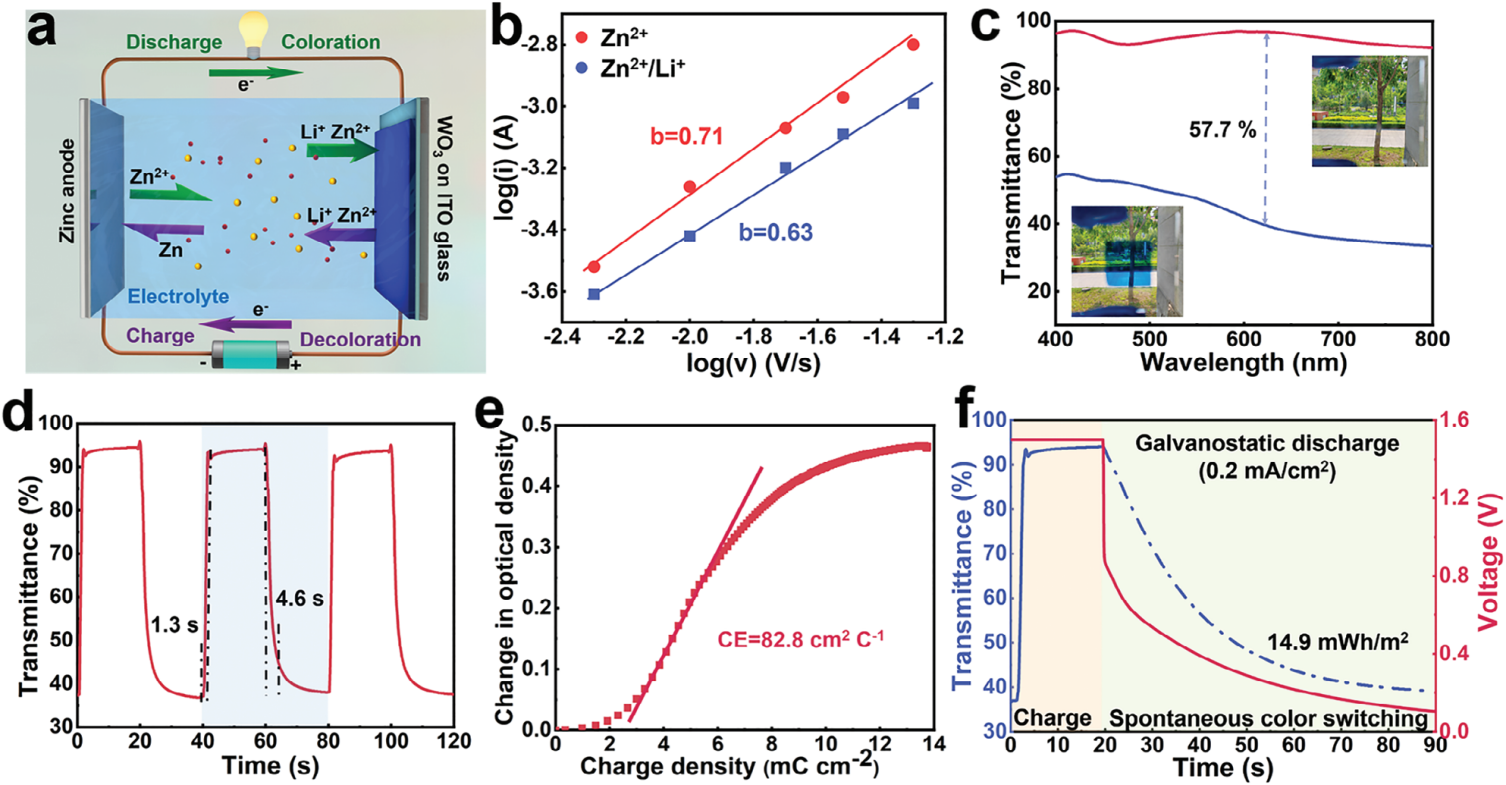
Electrochemical and electrochromic performance of the WO_3_ electrode. a) Schematic diagram of the operational principle of the Zn‐WO_3_ electrochromic device. b) Log (i) as a function of log (*v*) for the WO_3_ electrode in different electrolytes. The i refers to the currents of the anode peaks shown in Figure S14 (Supporting Information). The *v* represents the CV scan rate. The slopes of the lines determine the b values. c) Optical transmittance spectra of the WO_3_ electrodes at varying applied voltages (The red line and blue line represent 1.5 and 0.1 V, respectively). Inset: corresponding digital photos of the WO_3_ electrode. d) Real‐time transmittance spectrum of the WO_3_ electrode at 633 nm in the 0.1–1.5 V window. e) Changes in optical density at a light wavelength of 633 nm as a function of charge density. f) Transmittance of the WO_3_ electrode at the wavelength of 633 nm during the chronoamperometric charging process (solid blue line) and the galvanostatic discharge process at a current density of 0.2 mA cm^−2^ (dashed blue line). The red line is the corresponding potentiostatic charge and galvanostatic discharge curve.

With the careful design of the Zn^2+^/Li^+^ EG/PAAm photochromic hydrogel electrolyte, the fabrication of the WO_3_ cathode plays a vital role in constructing Zn‐EG/PAAm‐WO_3_ electro‐ and photo‐dual‐responsive chromatic devices. Electrodeposition is a strategy employed to produce a thin coating that facilitates control over morphology and offers excellent scalability.^[^ ^30^ ^]^ One of the pioneering manufacturers, Gesimat from Germany, utilized the electrodeposition technique to fabricate their large‐area dynamic window products.^[^ ^31^ ^]^ Herein, we adopted the well‐developed pulsed electrodeposition approach to manufacture a 370 nm‐thick WO_3_ cathode (Figure S13a, Supporting Information; see Experimental Section for fabrication approach).^[^ ^32^ ^]^ Notably, this pulsed electrodeposition approach provides WO_3_ nanoparticles of optimal size for efficient ion diffusion, leading to excellent electrochemical performance.^[^ ^2^, ^33^ ^]^ The X‐ray diffraction (XRD) pattern of the electrodeposited WO_3_ film, depicted in Figure S13b (Supporting Information), reveals its amorphous nature.^[^ ^30^, ^34^ ^]^ To investigate the electrochromic performance of the WO_3_ electrode, a two‐electrode configuration was employed, utilizing zinc as the anode and the WO_3_ electrode as the cathode (**Figure** **3**a). The designated electrolyte was prepared by dissolving 0.1 M Zn(ClO_4_)_2_ and 0.5 M LiClO_4_ in deionized (DI) water, ensuring optimal performance.

To clarify the role of Li^+^ in the coloration process of the WO_3_ electrode, we investigated the electrochemical kinetics of the WO_3_ electrode in Zn^2+^ electrolyte and Zn^2+^/ Li^+^ hybrid electrolyte. CV spectra of the WO_3_ electrodes were measured in the voltage range between 0.1 to 1.5 V at scanning rates from 5 to 50 mV s^−1^ (Figure S14, Supporting Information). The peak current i obeys a power‐law relationship (1) with the scan rate ν in the CV curves:^[^ ^35^ ^]^

(1) $$i=av^{b}$$

The charge storage mechanism can be clarified by the value of b determined from the slope of the plot of log (i) as a function of log (*v*). A b value of 0.5 suggests that the peak current shows a linear proportion with the square root of the scan rate, consistent with conventional diffusion‐controlled electrochemical processes. On the other hand, a value of b for 1 means the current is proportional to the scan rate, indicating a surface‐capacitive controlled mechanism.^[^ ^36^ ^]^ The b values for the WO_3_ electrode in Zn^2+^ and Zn^2+^/ Li^+^ electrolytes were determined to be 0.71 and 0.63 (Figure 3b), respectively. The addition of Li^+^ in the electrolyte led to a decrease in the value of b, indicating that Li^+^ promoted the diffusion behavior and enabled the intercalation of Li^+^.

The X‐ray photoelectron spectroscopy (XPS) results reveal that the color switching of the WO_3_ electrode originated from the oxidation and reduction of W (Figure S15, Supporting Information). Figure 3c shows the change in optical transmittance of the WO_3_ cathode measured in the aqueous electrolyte (i.e., Zn^2+^/ Li^+^ electrolyte). The WO_3_ cathode exhibits an optical contrast (△T) of 57.7% at 633 nm, which is higher than that measured in the propylene carbonate (PC)‐based electrolyte (52.1%, Figure S16a, Supporting Information) and higher than previous reports.^[^ ^12^, ^37^ ^]^ Due to the enhanced ionic conductivity facilitated by aqueous electrolytes, the coloration (t_c_) and bleaching (t_b_) times of the WO_3_ electrode in the aqueous electrolyte are 4.6 and 1.3 s respectively (Figure 3b), which surpasses that of the WO_3_ electrodes in the PC‐based electrolyte (t_c_ = 4.9 s, t_b_ = 1.7 s, Figure S16b, Supporting Information).

Another important metric for electrochromic electrodes is the coloration efficiency (CE). As shown in Figure 3e, the WO_3_ electrode exhibits an exceptional CE, with values reaching 82.8 cm^2^ C^−1^ at 633 nm, surpassing the CE tested in PC‐based electrolyte at 633 nm (Figure S16c, Supporting Information). This improvement is attributed to the rapid ion transport efficiency in aqueous electrolytes. The round‐trip energy efficiency is also crucial for the zinc anode‐based electrochromic platform. Through a chronoamperometry profile (by applying a 1.5 V voltage for 20 s), the calculated input energy consumed in bleaching the WO_3_ electrode is 24.03 mWh m^−2^ (Figure S17, Supporting Information), while the galvanostatic discharge process at a current density of 0.2 mA cm^−2^ retrieves 14.9 mWh m^−2^ along with a spontaneous coloration process (Figure 3f). Herein, the round‐trip energy efficiency is calculated to be 62%, which is superior to the state‐of‐the‐art reports.^[^ ^2^, ^10^ ^]^ Notably, while the electrochromic metrics of the WO_3_ electrodes in aqueous electrolytes surpass those tested in the PC‐based electrolyte, the cycling stability remains a significant challenge due to the dissolution issues of WO_3_ in aqueous electrolytes.^[^ ^38^ ^]^ As shown in Figure S18 (Supporting Information), the optical contrast of the WO_3_ electrodes in the aqueous electrolyte is maintained for only a few cycles before destructive decay occurs, whereas in the PC‐based electrolyte, the WO_3_ electrodes retain 87.9% of their initial optical contrast after 1000 cycles. This disparity is attributed to the presence of free water molecules, which disrupt the cycling performance of the WO_3_ electrodes.^[^ ^38^ ^]^ The PAAm hydrogel electrolyte, which minimizes free water molecules, is expected to greatly boost the cycling performance of the device while retaining excellent electrochromic metrics.

### 2.4 Rigid Zn‐WO_3_ Dimmer for Dynamic Windows

**Figure 4:**
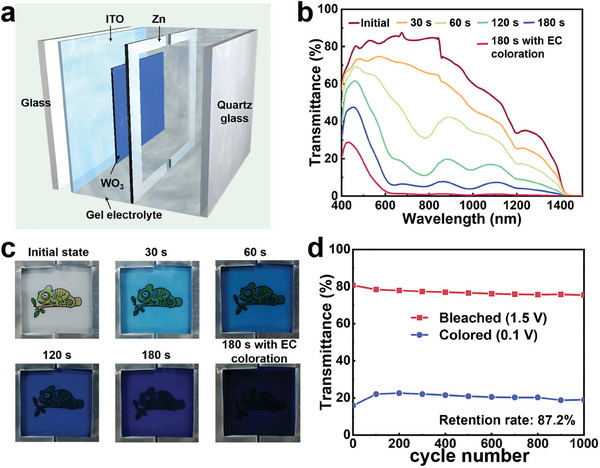
Electrochromic and photochromic performance of the Zn‐WO_3_ dimmer. a) Schematic configuration of the prototype Zn‐WO_3_ dimmer. b) Optical transmittance spectra of the Zn‐WO_3_ dimmer under different durations of sunlight exposure and the most tinted state by overlaying photochromic with electrochromic coloration effect. c) Corresponding digital photographs of the Zn‐WO_3_ dimmer under various tinted states. d) Cycling durability profile of the Zn‐WO_3_ dimmer under 1.5 and 0.1 V switching for 30 s interval.

To illustrate the dual‐responsive capabilities of the Zn‐WO_3_ dimmer, a 5 cm × 5 cm prototype device was constructed, as shown in **Figure** **4**a. The Zn^2+^/Li^+^ EG/PAAm hydrogel possesses outstanding photochromic properties and ionic conductivities at various temperatures, making it an excellent candidate electrolyte for Zn‐WO_3_ dimmers, serving as both a photochromic component and an electrolyte layer for ion transport. The top‐sealing quartz glass of the dimmer has superior transparency compared to ITO glass, not only providing high bleached transparency but also allowing sunlight transmission to trigger the photochromic effects. Herein, the Zn‐WO_3_ dimmer, utilizing Zn^2+^/Li^+^ EG/PAAm hydrogel as the electrolyte, exhibits a high transmittance contrast (ΔT = 83.1% at 633 nm) due to the synergy effect of photochromism and electrochromism, making the platform a promising candidate for dynamic windows. Figure 4b displays the synergy effect of photochromism and electrochromism, where the bleached device exhibits a transmittance of >80% over the visible light region (85.3% at 633 nm), superior to previous pioneering works.^[^ ^39^ ^]^ The high bleached transmittance is attributed to the high transparency of quartz glass as well as the Zn^2+^/Li^+^ EG/PAAm hydrogel electrolyte, which enhances the transmittance contrast tunability. However, the bleached transmission of the Zn‐WO_3_ dimmer is slightly lower than that of a single electrode (90.2% at 633 nm, Figure 3c). The incorporation of the Zn^2+^/Li^+^ PAAm hydrogel electrolyte leads to a reduction in transmittance at the device level. This Zn‐WO_3_ dimmer can be gradually switched to a colored state with 6% light transmittance at 633 nm when exposed to sunlight for 180 s. To further lower the colored transmittance, a voltage of 0.1 V was applied between Zn and WO_3_ electrodes. Thus, a tinted transmittance of 1.4% at 633 nm is achieved, providing better privacy for dynamic windows. Figure 4c reveals the coloration process of the Zn‐WO_3_ dimmer via photochromic and electrochromic effects, where the cartoon logo can be fully blocked at the lowest tinted transmittance, further confirming the good privacy provided by combining the photochromic and electrochromic effects within a single platform. Moreover, the use of Zn^2+^/Li^+^ EG/PAAm hydrogel as the electrolyte in the Zn‐WO_3_ dimmer not only significantly enhances electrochromic cycling stability but also preserves rapid switching times. Figure 4d shows that the Zn‐WO_3_ dimmer prototype retains 87.2% of its initial optical contrast after 1000 cycles, significantly superior to the cycle performance of the Zn‐WO_3_ platform using the pure aqueous electrolyte (Figure S18a, Supporting Information). Notably, the degradation of the WO_3_ in aqueous electrolytes is attributed to the formation of polytungstates. Small water molecules can attack the W‐O‐W bond and form two saturated W‐OH and HO‐W bonds, which causes a small local distortion of the lattice. Consequently, it becomes facile for WO_3_ in aqueous electrolytes to form tungsten and polytungstate ion structures, which are continuously generated during the coloration cycle.^[^ ^40^ ^]^ In contrast, the solvent in the EG/PAAm hydrogel electrolyte primarily consists of EG and water, with a major fraction of water being trapped by hydrogen bonds.^[^ ^25^ ^]^ Several factors prevent the degradation of the WO_3_: 1) It does not ionize as readily as free water; 2) Its molecular size is too bulky to penetrate the crystal lattice; 3) The bulky free radical groups of EG hinder the formation of dense or complex poly tungstate ionic structures.^[^ ^40^ ^]^ Consequently, the EG/PAAm hydrogel electrolyte ensures excellent cycling performance of the WO_3_ electrode. Figure S19 (Supporting Information) shows the electrochromic switching properties of the Zn‐WO_3_ dimmer prototype, which delivers rapid switching times (i.e., 8.1 s for coloration and 5.8 s for bleaching) in comparison with state‐of‐the‐art WO_3_‐based devices.^[^ ^30^, ^41^ ^]^

Additionally, the Zn‐WO_3_ dimmer delivers the most energy‐efficient strategy for electrochromic devices, as the emerging zinc anode‐based electrochromic devices are capable of retrieving consumed energy during the spontaneous coloration process.^[^ ^42^ ^]^ The bleached Zn‐WO_3_ dimmer possesses a potential difference between the Zn anode and WO_3_ cathodes, thus delivering an open‐circuit potential (OCP) of 1.11 V (Figure S20a, Supporting Information). To efficiently utilize the OCP of the Zn‐WO_3_ dimmer, an LED is connected to the dimmer through a “Joule thief circuit”. This setup vividly demonstrates the LED lighting up concurrently with the spontaneous coloration process (Figure S20b, Supporting Information). To quantitatively determine the energy retrieval functionality of the Zn‐WO_3_ dimmer, chronoamperometry charging and galvanostatic discharging profiles are used to calculate the input energy and retrieved energy, respectively. The chronoamperometric charging was conducted by applying a voltage of 1.5 V to the Zn‐WO_3_ dimmer for 10 s, while the galvanostatic discharging was conducted at a current density of 0.35 mA cm^−2^. The duration of 10 s was selected because this time is sufficient to fully bleach the dimmer, while the current density of 0.35 mA cm^−2^ enables a rapid coloration process. It was calculated that the input energy during a round trip is 57.8 mWh m^−2^ (Figure S21a, Supporting Information), while the retrieved energy is 37.8 mWh m^−2^ (Figure S21b, Supporting Information). Consequently, the round‐trip energy efficiency of the Zn‐WO_3_ dimmer is 65.40%, which is better than previous pioneering reports.^[^ ^2^, ^10^ ^]^

These outstanding metrics collectively demonstrate that the Zn‐WO_3_ dimmer represents an intriguing potential technology whose future impact on dynamic windows may be substantial. Remarkably, such dynamic windows have good adaptability for use in high latitude regions, as the Zn^2+^/Li^+^ EG/PAAm hydrogel can also function at −40 °C.

### 2.5 Flexible Zn‐WO_3_ Dimmer for Augmented Reality Smart Glasses

The Zn‐WO_3_ dimmer offers significant advantages for wearable augmented reality smart glasses (ARSGs) due to its high bleached transparency and large transmittance contrast. ARSGs require energy efficiency and lightweight design for prolonged wear, the rigid Zn‐WO_3_ platform no longer meets these requirements. Therefore, a flexible design was introduced using the excellent mechanical properties of the Zn^2+^/Li^+^ EG/PAAm hydrogel. This design utilizes a flexible ITO‐coated polyethylene terephthalate (PET) membrane as a substrate to reduce weight and enhance flexibility.

**Figure 5:**
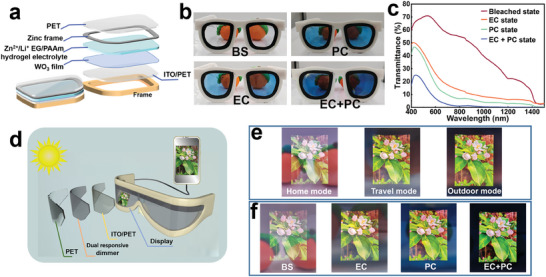
Flexible Zn‐WO_3_ Dimmer for Augmented Reality Smart Glasses. a) Schematic illustration of the Zn‐WO_3_ dimming sunglasses. b) Digital photographs of the Zn‐WO_3_ dimming sunglass in bleached state (BS), electrochromic state (EC), photochromic state (PC), and state overlaying electrochromic and photochromic effects (EC+PC). c) Transmittance spectra of the Zn‐WO_3_ dimming sunglass at different tinted states. d) Schematic illustration of the Zn‐WO_3_ dimmer integrated with AR glasses. e) Photographs taken through the three electrochromic dimming levels of the XREAL Air 2 Pro AR glass and f) four dimming levels of Zn‐WO_3_ dimmer enhanced XREAL Air 2 Pro AR glass.

**Figure** **5**a schematically illustrates the structure of the flexible Zn‐WO_3_ dimmer, where the Zn anode can be integrated into glass frames to increase glass mechanical strength and reduce weight. Figure 5b,c depict digital photographs of the flexible Zn‐WO_3_ dimmer at different tinted states and their corresponding transmittance spectra. When exposed to sunlight for 300 s (PC state), the dimmer becomes semi‐transparent with a transmission of 13.8% at 633 nm. Additionally, the flexible Zn‐WO_3_ dimmer can achieve a transmission of 22.3% at 633 nm (EC state) by applying a voltage of 0.1 V for 60 s to induce the electrochromic effect. Combining the photochromic (PC) and electrochromic (EC) effects (EC+PC state) reduces the dimmer's transmission to 4.7% at 633 nm, surpassing state‐of‐the‐art WO_3_‐based flexible electrochromic devices.^[^ ^13^, ^43^ ^]^

As a key developmental direction in next‐generation display technology, VR displays effectively expand the field of view while minimizing external distractions. As illustrated in Figure S22a (Supporting Information), images in a VR system are typically generated by a display module measuring 2–3 inches in size. The light emitted from the display module is transformed into a virtual image that occupies the entire field of view (FOV) using specially designed magnifying lenses. Furthermore, VR technology simulates depth cues for 3D virtual objects by presenting distinct images to each eye. The EC+PC state of the Zn‐WO_3_ dimmer can nearly completely block external light, offering users a fully immersive virtual experience. Figure S22b (Supporting Information) shows a general structure diagram of an optical perspective AR system, where the optical combiner is the most critical component, as it must display accurate depth cues that align with the real world. However, bright outdoor environments present a challenge for image depth accuracy due to ambient light. The incoming background light can be intelligently adjusted by the EC state of the Zn‐WO_3_ dimmer, adjusting the virtual image's depth cues in response to the background light.^[^ ^44^ ^]^ Meanwhile, ambient light can trigger the photochromic effect, facilitating a seamless transition from AR to opaque VR glasses.

To compare the performance of the Zn‐WO_3_ dimmer in ARSGs with leading commercial ARSGs equipped with electrochromic lenses (i.e., XREAL Air 2 Pro AR Glasses), we attached our flexible Zn‐WO_3_ dimmer on top of the XREAL Air 2 Pro AR glasses (Figure 5d). While the XREAL Air 2 Pro AR glasses can instantly switch between three electrochromic dimming levels (home, travel, and outdoor modes, Figure 5e), the Zn‐WO_3_ dimmer enables four dimming levels by overlaying both electrochromic and photochromic effects (Figure 5f). These four dimming levels enhance adaptability to various lighting conditions, ensuring excellent visual performance of ARSGs. Moreover, the EC+PC state of the Zn‐WO_3_ dimmer provides a significantly darker background compared to the outdoor mode of the XREAL Air 2 Pro AR glass, facilitating a seamless transition from AR to opaque VR glasses. These performance attributes highlight the promising potential of flexible Zn‐WO_3_ dimmers for ARSGs.

## 3 Conclusion

In summary, we developed a highly transparent photochromic Zn^2+^/Li^+^ EG/PAAm hydrogel electrolyte for the first time. This photochromic hydrogel electrolyte demonstrates excellent ionic conductivity across a wide range of temperatures, as well as robust mechanical performance at low temperatures, making it an ideal candidate electrolyte for electrochromic devices intended for extreme conditions. Furthermore, the photochromic hydrogel electrolyte exhibits outstanding transmittance contrast (ΔT = 90.2% at 633 nm), enabling the fabrication of electro‐ and photo‐responsive chromatic devices with high‐contrast dimming capability. By integrating the Zn^2+^/Li^+^ EG/PAAm hydrogel electrolyte into well‐designed Zn‐WO_3_ electrochromic devices, the first dual‐responsive Zn‐WO_3_ dimmer is demonstrated. These dual‐responsive dimmers achieve impressive transmittance contrast (ΔT = 83.1% at 633 nm), surpassing state‐of‐the‐art electrochromic devices and showing great promise for dynamic windows regulating solar heat and daylight. Moreover, the excellent mechanical properties of the photochromic hydrogel electrolyte make it suitable for flexible Zn‐WO_3_ dimmers, which can enhance visual performance in AR glasses by offering more intermediate color states and a nearly opaque state. These advancements address concerns about low transmittance contrast in electrochromic devices, representing a significant step forward for high‐contrast dimming in dynamic windows and AR glasses.

While the development of this futuristic Zn‐WO_3_ dimmer is on the drawing board, further efforts are needed from the electrochromic and photochromic communities to advance this technology from the laboratory to practical applications. The requirements for light‐weight wearable devices can be satisfied by further improving the electrochromic response of the Zn‐WO_3_ dimmer. In comparison to the electrodeposited WO_3_ electrochromic layer,^[^ ^30b^ ^]^ WO_3_ nanorods and WO_3_ nanodots can be selected to construct electrochromic electrodes with faster response, as they accelerate electron transfer dynamics and increase the active surface area.^[^ ^45^ ^]^ Furthermore, the relatively slow response of the Zn‐WO_3_ dimmer is attributed to the reduced cation conductivity in the Zn^2+^/Li^+^ PAAm hydrogel electrolyte. Therefore, increasing the concentration of salts (LiClO_4_, Zn(ClO_4_)_2_) in the electrolyte may enhance its conductivity. Additionally, the zinc anode frame generates a voltage drop across large‐area devices during operation, which can impact the switching rate.^[^ ^16^ ^]^ A highly transparent zinc grid can help create a uniform and stable electric field, improving the switching performance.^[^ ^16^ ^]^ Currently, the photochromic hydrogel's discoloration process takes 5 h, which must be reduced to match the speed of commercial photochromic sunglasses for broader practical use. To this end, we have reviewed relevant photochromic literature to identify effective methods for reducing the bleaching time of Zn^2+^/Li^+^ PAAm hydrogels (Table S3, Supporting Information). As shown in Table S3 (Supporting Information), heating methods are employed to accelerate the oxidation of WO_3_, thus reducing the bleaching time. Additionally, a dark environment can inhibit the electron transfer process induced by light excitation in WO_3_, thereby facilitating its oxidation. Oxidative atmospheres, such as ozone, can also enhance the bleaching of the colored hydrogel electrolyte. Furthermore, adjusting the bleaching times of the photochromic hydrogel electrolyte can potentially be achieved by incorporating Cu ions into the hydrogel for faster discoloration processes.^[^ ^7a^ ^]^ While the Zn^2+^/Li^+^ EG/PAAm hydrogel electrolyte exhibits excellent water retention capabilities, its long‐term stability is impacted by water loss. Addressing this challenge through improved sealing methods for the photochromic hydrogel electrolyte is crucial for real‐world applications. With all these aforementioned challenges addressed, real‐world applications of the dual‐responsive Zn‐WO_3_ dimmer are on the near horizon.

## 4 Experimental Section

### Materials

All the chemicals were of analytical grade and were used without further purification. MBAA, AAm, TEMED, APS, Zn(ClO_4_)_2_ and Zinc foil (Zn, 99.9%) were purchased from Macklin Biochemical Technology Co. Ltd. Tungsten flakes were obtained from Yungbiao Metal Material Co. Ltd. EG were sourced from Beede Pharma. Hydrochloric acid (HCl) and LiClO_4_ were purchased from Sinopharm Chemical Reagent Co. Ltd. Propylene carbonate was obtained from Shanghai Yi En Chemical Technology Co. Ltd. Hydrogen peroxide solution (H_2_O_2_, 30 wt.%) and perchloric acid (HClO_4_, 70%) was obtained from Tianjin Beilian Fine Chemicals Development Co. Ltd. Sodium tungstate dihydrate (Na_2_WO_4_·2H_2_O, ≥99%) was purchased from Aladdin. ITO coated PET, PET membrane, ITO coated glass, and quartz glass were purchased from Zhuhai Kaivo Glass Co. Ltd.

### Preparation of Amorphous WO_3_ Nanodots

The amorphous WO_3_ nanodot colloids were synthesized using this previously reported methodology, which combines reagent‐free electrophoretic synthesis with a subsequent decomposition process.^[^ ^12^ ^]^ In a typical procedure, two tungsten sheets were placed parallel to each other in a beaker filled with a diluted solution of HCl (0.02 M). A direct current (DC) bias of 40 V was then applied across the tungsten sheets for 30 min, resulting in the formation of crystalline hydrated WO_3_ particles. These particles were subsequently collected via centrifugation and dispersed in a solvent mixture consisting of 8 mL of EG and 4 mL of DI water. Finally, the crystalline hydrated WO_3_ particles were decomposed into amorphous WO_3_ nanodot colloid by heating at a temperature of 95 °C for 30 min.

### Preparation of EG/PAAm Hydrogel Electrolytes

First, Li(ClO_4_) (1.6 g) and Zn(ClO_4_)_2_ (0.37 g) were dissolved in 5 mL of the as‐prepared WO_3_ nanodot colloid (with an EG to water ratio of 2:1). Then, AAm monomer (1.6 g), MBAA (13 mg), and (NH_4_)_2_S_2_O_8_ (26 mg) were added to the solution. The mixture was subjected to sonication and agitation until a clear solution was achieved. Following this, 5 µL of TEMED was added to the solution to form the precursor. The precursor was then immediately poured into a plastic mold and thermally treated at 40 °C for 20 min to fabricate the EG/PAAm hydrogel electrolytes. Control hydrogel samples were prepared using the same procedure, except pure DI water was used and different slats were employed for comparison purposes.

### Preparation of Amorphous Electrochromic WO_3_ Cathode

The amorphous electrochromic WO_3_ cathode was prepared using a pulsed electrodeposition method.^[^ ^30^, ^45^ ^]^ ITO‐coated glass/PET substrates were sequentially sonicated with acetone, ethanol, and deionized water, then dried in ambient air. The electrodeposition procedure was conducted in a three‐electrode configuration with the ITO‐coated glass/PET (5 × 5 cm^2^) serving as the working electrode, Ag/AgCl electrode as the reference electrode, and a 5 × 5 cm^2^ stainless steel mesh as the counter electrode. The precursor solution was prepared by dissolving 0.412 g Na_2_WO_4_·2H_2_O in 100 mL DI water, followed by adding 0.8 mL HClO_4_ and 0.26 mL H_2_O_2_ under continuous magnetic stirring. The WO_3_ films were deposited by applying a pulsed potential of −0.7 V for 0.2 s followed by 0.1 V for 0.8 s. The pulsed electrodeposition process was repeated for 2000 cycles.

### Assembly of Zn‐WO_3_ Dimmer

The rigid Zn‐WO_3_ dimmer was assembled using a WO_3_‐coated ITO glass as the electrochromic cathode, the photochromic hydrogel electrolyte attached to the WO_3_ cathode as the electrolyte, and a zinc frame attached to quartz glass as the anode. The flexible Zn‐WO_3_ dimmer was prepared by using WO_3_‐coated ITO/PET as the cathode and a zinc frame attached to PET as the anode, with all other parameters being identical.

### Characterization

The crystal structures, morphology, and optical properties of the samples were examined by X‐ray diffraction (XRD, Rigaku D/Max 2500 PC diffractometer with a graphite monochromator and Cu Kα radiation (λ = 0.15418 nm), field emission scanning electron microscopy (FE‐SEM) (Quanta 250 FEG), high‐resolution transmission electron microscope (HRTEM, FEI Talos f200s), and UV–vis‐NIR spectrophotometry (UH5700). The anti‐drying property of the EG/PAAm hydrogel was assessed using thermogravimetric analysis (TGA) conducted on a Netzsch STA 449 C instrument under airflow, with a heating rate of 10 °C min^−1^. The mechanical property of the EG/PAAm hydrogel was tested using in‐situ electronic universal testing machine (Shimadzu AGS‐X‐10 kN) at −20, −40, 50, and 80 °C. Infrared spectroscopy tests for the hydrogels and colloids were conducted using a Fourier transform infrared spectrometer (FTIR, Thermo Fisher Scientific Nicolet iS5). Imaging tests of the dimmers were performed using the XREAL Air 2 Pro AR glass. All electrochemical measurements were carried out using an electrochemical workstation (CHI‐760E; CH Instruments, Shanghai, China) in a two‐electrode configuration. The WO_3_ electrode served as the working electrode, and Zn acted as both the counter electrode and reference electrode. In‐situ optical transmittance measurements of single electrodes were conducted in a quartz cuvette recorded by UH5700 spectrometer. ITO glass in the electrolyte served as the baseline for measuring the electrochromic performance of the single electrodes, while ambient air was used as a baseline for measuring the devices’ overall electrochromic performance.

## Conflict of Interest

The authors declare no conflict of interest.

## Supporting information



Supporting Information

## Data Availability

The data that support the findings of this study are available from the corresponding author upon reasonable request.
