# Supplementary material for: Electro‐ and Photo‐ Dual Responsive Chromatic Devices for High‐Contrast Dimmers
Source: Adv Mater. 2024 Dec 30;37(7):2410703. doi: 10.1002/adma.202410703 (PMC11837896; doi:10.1002/adma.202410703)
Supplement: Supplementary file 1 — Supporting Information [file ADMA-37-2410703-s001.docx]

Supporting Information

Electro- and photo- dual responsive chromatic devices for high-contrast dimmers

Bin Wang, Pengcheng Liu, Feifei Zhao, Bingkun Huang, Wu Zhang^*^, Abdulhakem Y. Elezzabi, Linhua Liu, William W. Yu^*^, and Haizeng Li^*^

**Table S1. Comparison of Gel Electrolytes used in Electrochromic Devices**

| **Ref.** | **Active material** | **Electrolyte matrix/physical state** | **Integrated performance** | **Working temperature (^o^C)** | **Application** |
| --- | --- | --- | --- | --- | --- |
| [1] | PEDOT | Butyl acrylate/Solid | Thermotropic, Electrochromic | −25~50 | Window |
| [2] | Viologen derivatives | PVB/Liquid | Solar cell-powered | ~ | Window |
| [3] | APV@TiO_2_ /PB | NIPAM/Solid | Thermotropic, Electrochromic | ~ | Window |
| [4] | WO_3_ | Polyacrylate  /Solid | Viscosity | ~ | Flexible devices |
| [5] | WO_3_/NiO | IOBA and DEEA/Solid | ~ | −40~150 | Display |
| [6] | Ethyl viologen | bisgluconamide molecules/quasi-solid | extreme temperature-tolerant | −196~200 | Flexible devices |
| [7] | WO_3_/NiO | UV-light adhesives/Solid | ~ | ~ | Dimmer |
| **This work** | WO_3_ films/ WO_3_ nanodots | EG-WO_3_ nanodots PAAm/Solid | Photochromic, Electrochromic  Viscosity | −40~40 | Window, Dimmer |

The comparison in Table S1 shows that most gel electrolytes function primarily as ion conductors in electrochromic devices. In contrast, this work introduces a novel approach where WO_3_ nanodots are employed as photochromic active materials in the design of gel electrolytes. By incorporating this photochromic hydrogel electrolyte into Zn-WO_3_ electrochromic devices, the first electro- and photo-dual responsive chromatic device is created for high-contrast dimmers.

**Table S2. Comparison of Current State-of-the-Art Electrochromic Devices.**

| **Ref.** | **material** | **t_b_/t_c_**  **(s)** | **ΔT**  **（nm）** | **Bleached transmittance (%)** | **Cycle stability** |
| --- | --- | --- | --- | --- | --- |
| [8] | Zn/WO_3_ | 10.3/2.7 | 79 % at 632.8  nm | ~78 | 200 cycles (43 %  optical loss) |
| [9] | h-Cs_0.32_WO_3_ | 105.5/7.6 | 65 % at 633  nm | ~65 | ~ |
| [10] | AP-WO_3_ | 7.2/6.4 | 60.7 % at 633  nm | ~80 | 1000 cycles (60.2 % capacity retention) |
| [11] | TiO_2-x_ | At least 20 | 74.5 % at 633  nm | ~75 | 500 cycles (89.8 % capacity retention) |
| [12] | Zn/V_3_O_7_ | 30.4/12 | 19 % at 632.8  nm | ~68 | ~ |
| [3] | APV@TiO_2_/PB | 0.6/0.82 | ~40 % at 605  nm | ~85 | 6000 s (no degradation) |
| [13] | PHPA/PMMA film | 24.3 | 80.1 % at 501  nm | ~90 | 500 cycles (2 %  optical loss) |
| [14] | Nb_18_W_16_O_93_ | 40.4/12.9 | 77 % at 633  nm | 80.5 | ~ |
| [15] | NiMoO_4_ | 30/20 | 65.9 % at 480  nm | ~75 | ~ |
| **This work** | WO_3_ | 5.8/8.1 | 83.1 % at 633  nm | 85.3 | 1000 cycles (12.8 %  optical loss) |

As shown in Table S2, in comparison with previous reports, the Zn-WO_3_ dimmer exhibits superior performance parameters compared to inorganic oxide-based ECDs. However, its switching time is slower than that of viologen-based ECD (APV@TiO_2_/PB). Future advancements are anticipated to produce inorganic electrochromic materials capable of achieving faster switching time (< 1s).


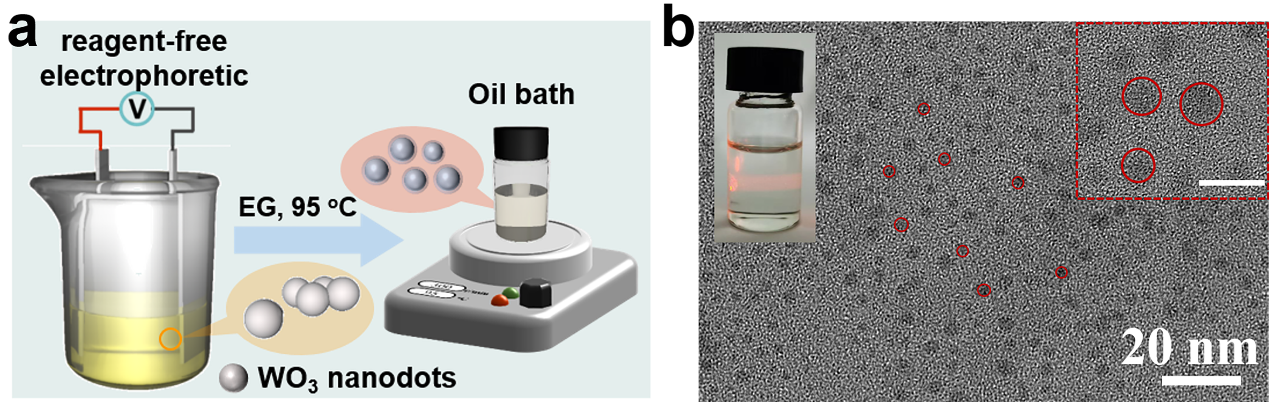


**Figure S1.** Preparation of EG-capped WO_3_ nanodots. a) A schematic represents the process employed in producing ultrasmall WO_3_ nanodots. b) TEM images of ultrasmall WO_3_ nanodots. The inset digital photograph illustrates the WO_3_ nanodot dispersion with a concentration of 1 mg/mL.

As shown in Figure S1a, the amorphous WO_3_ nanodots were synthesized via a reagent-free electrophoretic technique, followed by a decomposition process in EG solvent^[16]^. Figure S1b shows the transmission electron microscopy (TEM) images of the ultrasmall WO_3_ dots, confirming the effective preparation of WO_3_ dots through using an EG-based solvent. The size of these WO_3_ dots ranges from 2 to 5 nm, accompanied by a distinct Tyndall phenomenon when being dispersed in DI water (inset shown in Figure S1b)


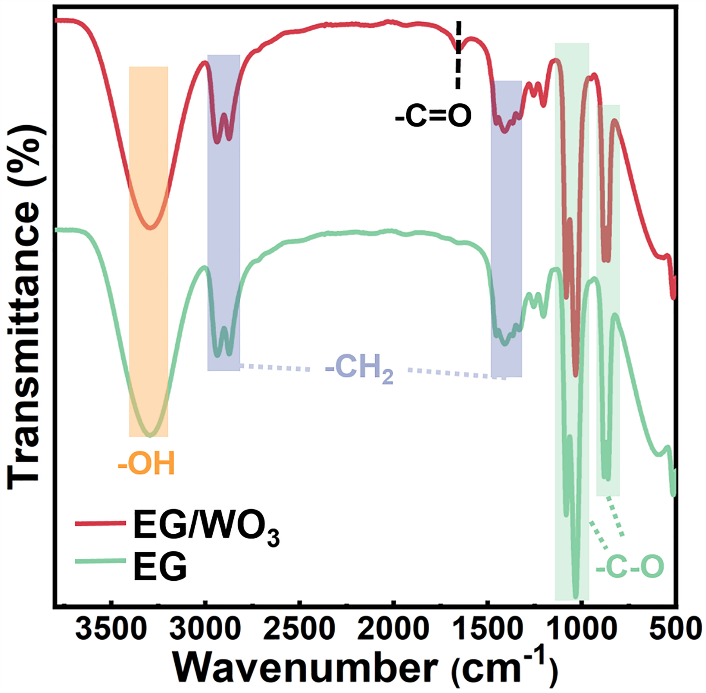


**Figure S2.** FTIR spectra of the pure EG and EG-capped WO_3_ colloid

As illustrated in Figure S2, the FTIR spectrum of pure EG shows several features, including the O–H group at 3292 cm⁻¹, the stretching vibrations of –CH_2_ at 2935 and 2873 cm⁻¹, and the bending vibrations of –CH_2_ within the range of 1455–1200 cm⁻¹, as well as the stretching and bending vibrations of -C–O at 1031 and 860 cm⁻¹, respectively^[17]^. On the other hand, the FTIR spectrum of EG-capped WO_3_ colloid exhibits a stretching vibration of C=O (1660 cm^-1^)^[18]^, indicating that EG undergoes a reaction to lose hydrogen atoms, which provides additional electrons and protons for WO_3_.


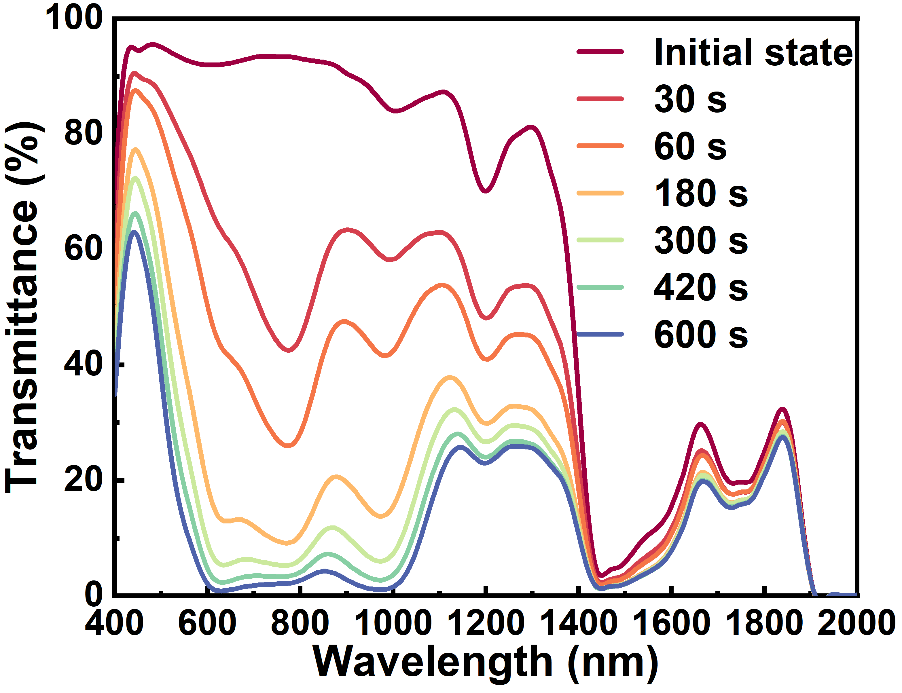


**Figure S3.** The transmission spectrum of the Zn^2+^/ Li^+^ PAAm hydrogel changes with the duration of light exposure.

As shown in Figure S3, the Zn^2+^/ Li^+^ PAAm hydrogel demonstrates a noticeable color change when exposed to sunlight within 30 s. Furthermore, after a light exposure duration of 600 s, the Zn^2+^/ Li^+^ PAAm hydrogel attains its maximum coloration, rendering it nearly opaque in the visible light range. The light regulation region spans both the visible and near-infrared spectra, achieving an optical contrast of 90.2 % at 633 nm and 47 % at 1200nm, respectively.


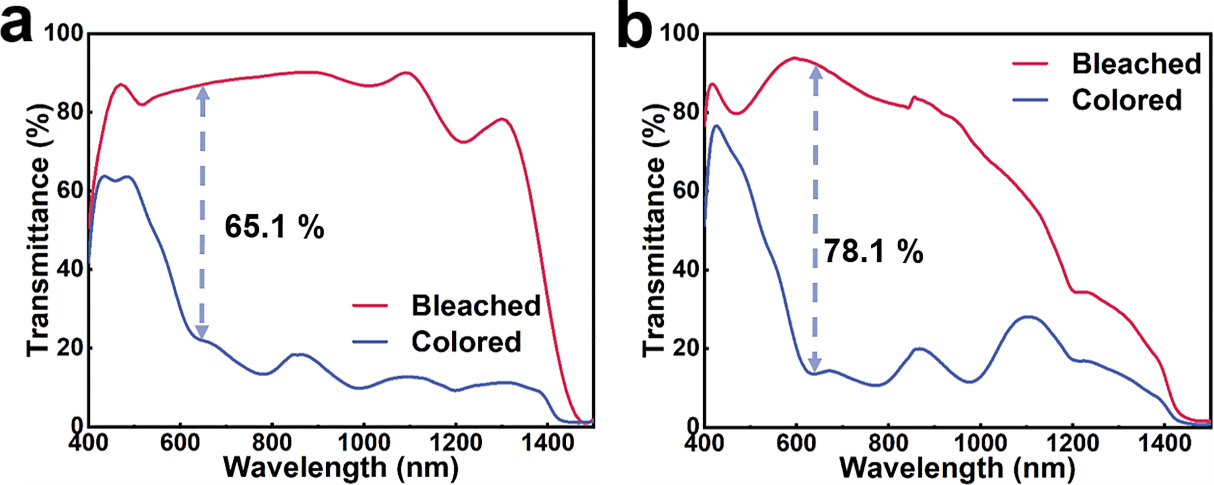


**Figure S4**. The optical transmittance spectra of the EG/PAAm hydrogel electrolyte with a) 0.6 M Zn(ClO_4_)_2_, b) 0.1 M Zn(ClO_4_)_2_ and 0.5 M Al(ClO_4_)_3_.

As shown in Figure S4a, the EG/PAAm hydrogel electrolyte containing pure Zn^2+^ exhibits the most inferior photochromic properties with an optical contrast of 65.1 % at 633 nm. The optical transmittance of the EG/PAAm hydrogel electrolyte containing Zn^2+^/ Al^3+^ is 13.7 % at 633 nm after 600 s of light exposure, and the optical contrast is 78.1 % at 633 nm. Its photochromic performance is also inferior to that of EG/PAAm hydrogel electrolyte containing Zn^2+^/ Li^+^ (Figure 3b).


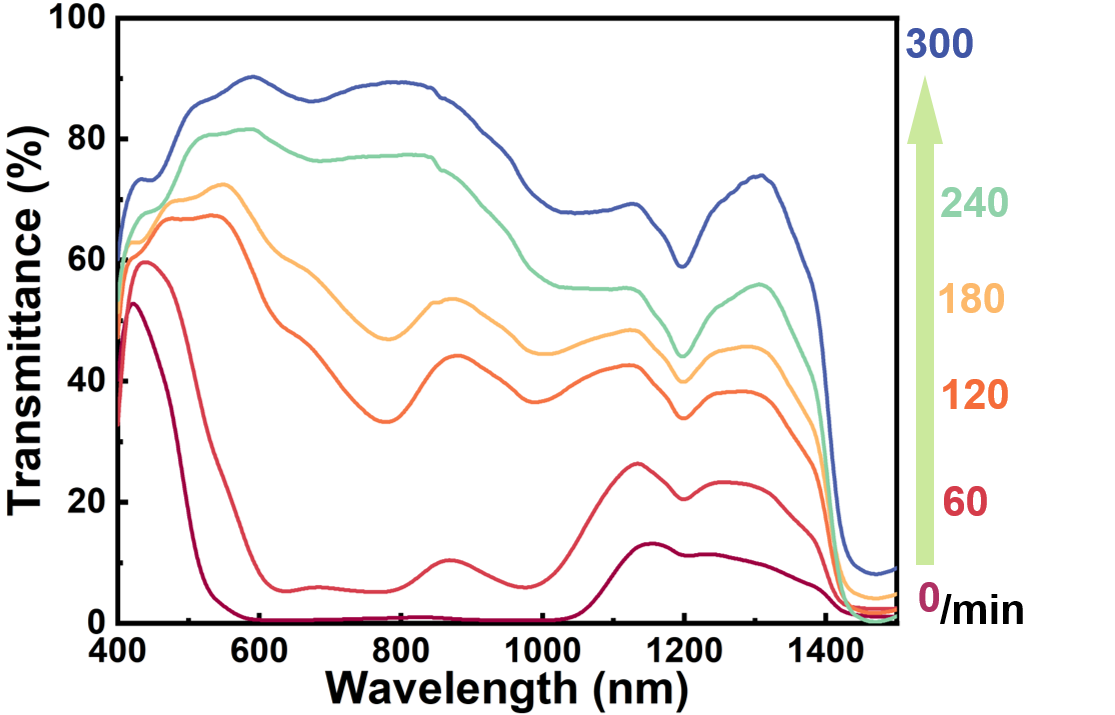


**Figure S5.** Transmittance spectra of the Zn^2+^/Li^+^ PAAm hydrogel during the bleaching process

As shown in Figure S5, in a darkroom environment, the Zn^2+^/Li^+^ PAAm hydrogel reached 55.8 % of its maximum transmittance within the first 2 hours; however, it required 5 hours to completely return to its initial state. Future advancements are expected to produce materials with improved efficiency in self-fading.


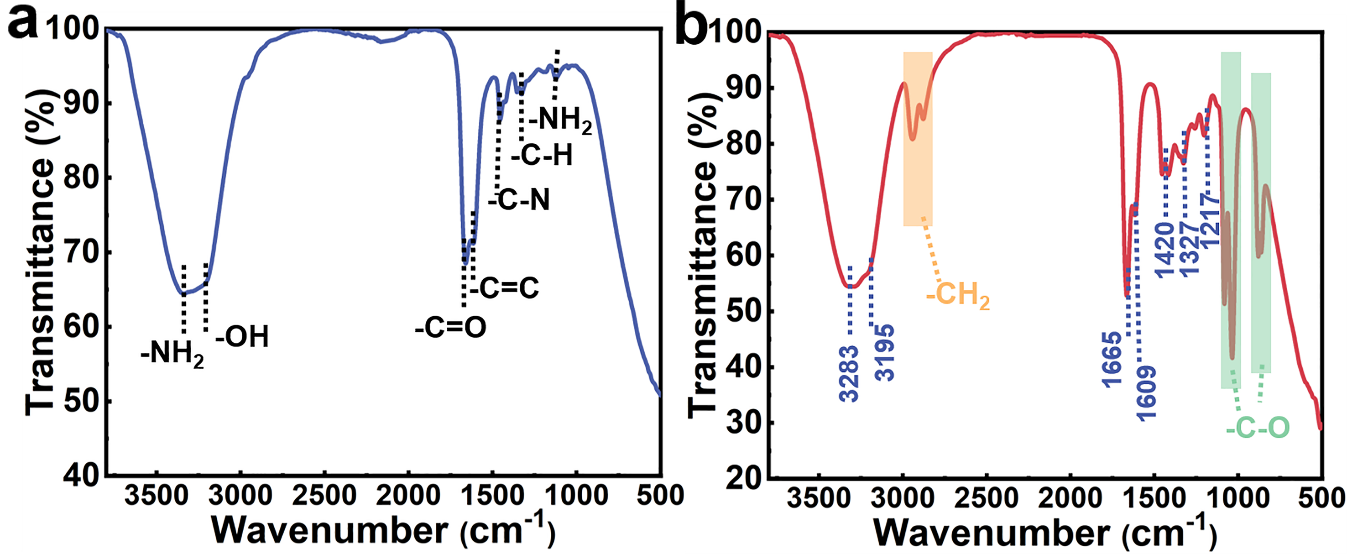


**Figure S6.** FTIR spectra of a) pure PAAm hydrogel and b) EG/PAAm hydrogel

As shown in Figure S6, the FTIR spectrum of pure PAAm hydrogel exhibits characteristic bands around 3342, 3227, 1660, 1611, 1455, 1326, and 1225 cm^-1^, which is ascribed to the symmetric stretching of -NH_2_, stretching vibration of -OH, stretching vibration of -C=O, stretching vibration of -C=C, -C-N stretching for primary amide, -C-H deformation, and -NH_2_ rocking, respectively^[19]^. In addition to the characteristic peaks of PAAm, the FTIR spectrum of EG/PAAm hydrogel also shows the peaks associated with EG, such as the bands at 2940 and 2880 cm^-1^ that are ascribed to the stretching vibration of -CH_2_, and the bands at 1323 and 996 cm^-1^ that are ascribed to the stretching and bending vibration of -C-O, respectively^[17]^.


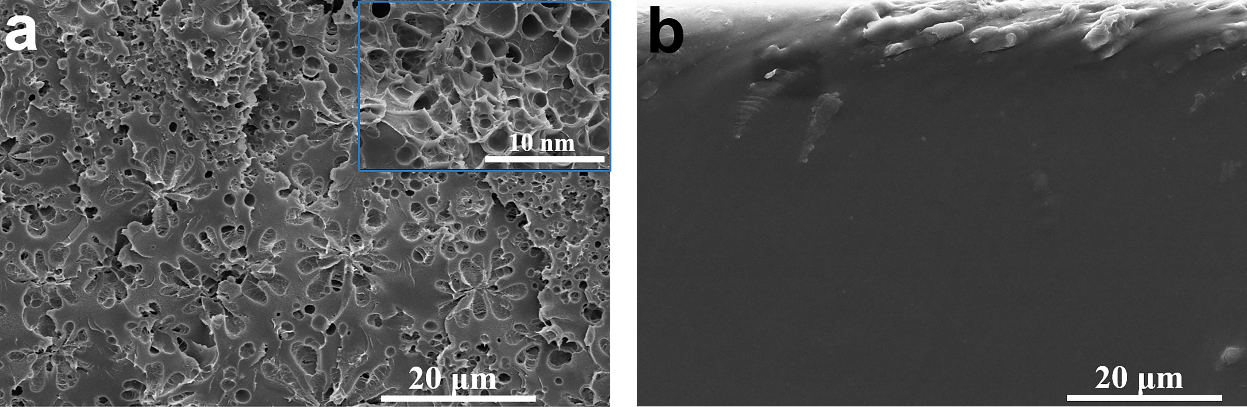


**Figure S7**. The cross-sectional SEM images demonstrate the microstructure of the hydrogels. a) The cross-sectional SEM images of the freeze-dried pure PAAm hydrogel. b) The cross-sectional SEM image of the freeze-dried EG/PAAm hydrogel.

Figure S7a presents the SEM images of the freeze-dried pure PAAm hydrogel, exhibiting a homogeneous porous network with pore sizes ranging from 2 to 10 μm. This uniform porosity is attributed to the sublimation of unbound water during lyophilization. In contrast, the EG/PAAm hydrogel exhibits a dense structure with a flat morphology after being freeze-dried at -50 ^o^C for 120 hours (Figure S7b). This suggests an interaction between EG and the hydroxyl groups in water molecules, effectively reducing the volume of free water and resulting in less sublimation during lyophilization.


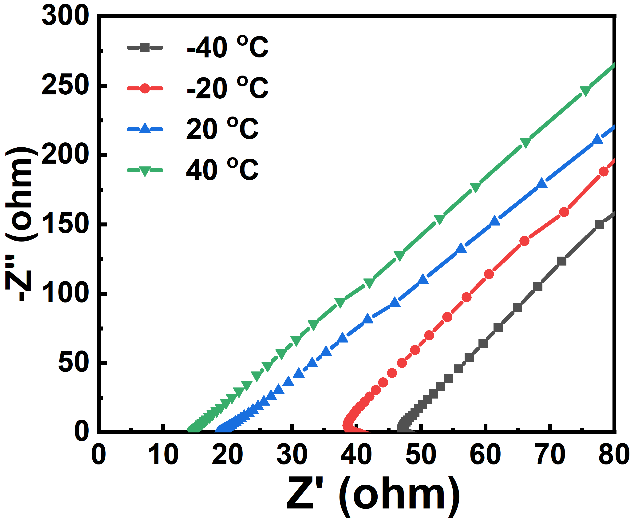


**Figure S8**. EIS curves of the EG/PAAm hydrogel at different temperatures.

As shown in Figure S8, the internal resistance of the EG/PAAm hydrogel is only 18.6 Ω at 20 ^o^C, but it gradually increases to 47.4 Ω at -40 ^o^C as the temperature decreases. The increase in temperature makes ion diffusion faster, resulting in an internal resistance of only 14.7 Ω at 40 ^o^C. The ionic conductivities of the hydrogels were derived using the equation σ = l/RA, where R is the measured impedance, and l and A are the thickness and cross-sectional area of the hydrogels, respectively.


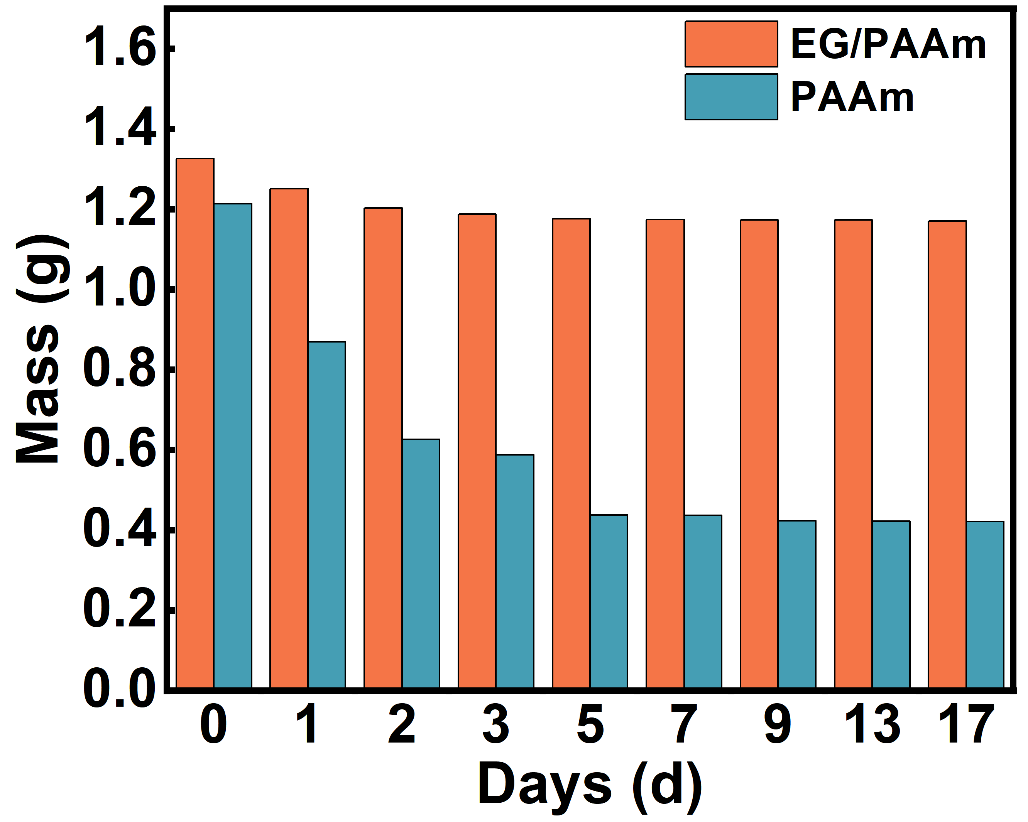


**Figure S9**. The changes in the mass of the EG/PAAm hydrogel and pure PAAm hydrogel within 17 days

As shown in Figure S9, the initial weight of the EG/PAAm hydrogel was 1.33 g. After 17 days, due to continuous water loss, its weight decreased to 1.17 g, and the water loss ratio was 11.8%. In comparison, the initial weight of the pure PAAm hydrogel was 1.21 g. After 17 days, its weight decreased to 0.42 g, resulting in a water loss ratio of 65.3 %.


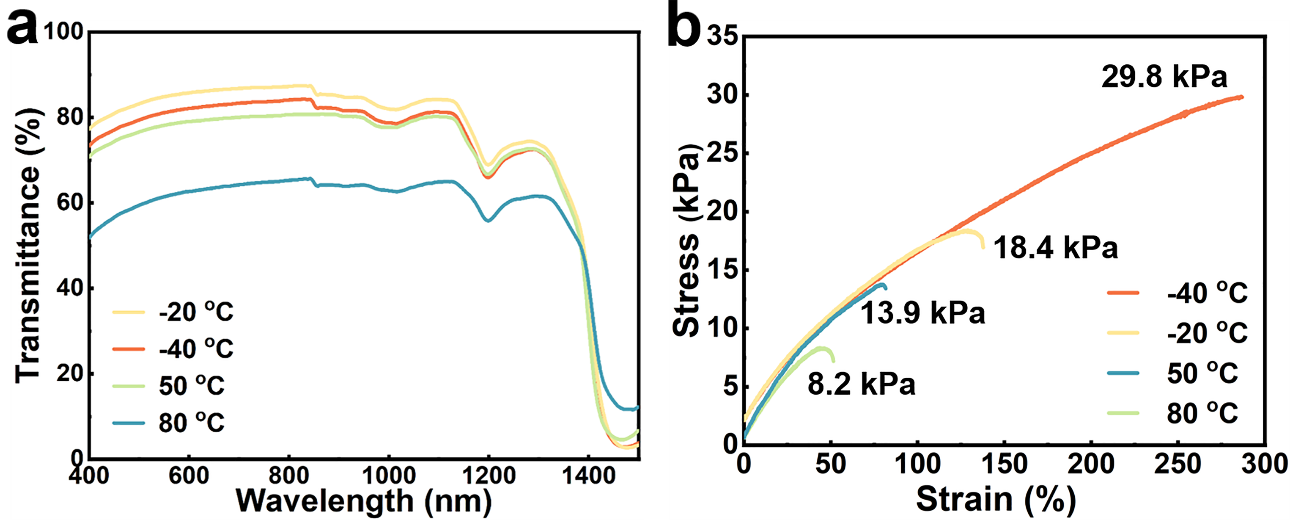


**Figure S10.** a) The optical transmittance spectra of the EG/PAAm hydrogel under different temperatures b) Stress-strain curves of the EG/PAAm hydrogel under different temperatures

As shown in Figure S10a, the EG/PAAm hydrogel's transmittance at temperatures of -20 ^o^C, -40 ^o^C, 50 ^o^C, and 80 ^o^C are 86,2 %, 82.6 %, 79.4 %, and 63.2 %, respectively. Even at the extreme conditions, the EG/PAAm hydrogel retains a high level of transmittance; however, its mechanical properties exhibit deterioration in high-temperature environments. As shown in the figure S10b, the tensile strength of the EG/PAAm hydrogel at 80°C is 8.2 kPa. Therefore, it is imperative to enhance the mechanical properties of the EG/PAAm hydrogel to fulfill its requirements across different application scenarios


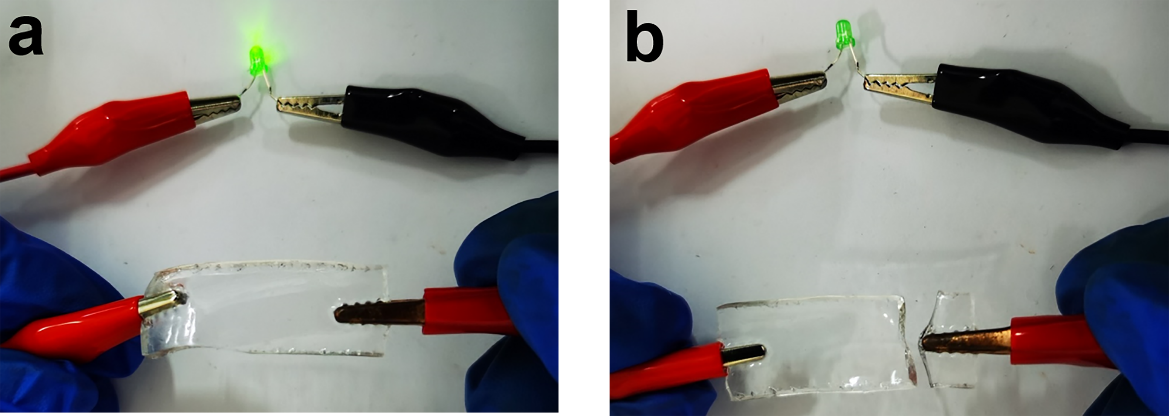


**Figure S11**. Stretchability of pure PAAm hydrogel electrolyte. a) The initial state of pure PAAm hydrogel electrolyte. b) The pure PAAm hydrogel electrolyte is easily fractured by applying a tiny tensile force.

Figure S11a demonstrates the initial state of the pure PAAm hydrogel electrolyte. It has excellent conductivity and can conduct electricity to light up an LED. However, the pure PAAm hydrogel electrolyte breaks and loses its electrical conductivity when stretched (Figure S11b). The poor stretchability of the pure PAAm hydrogel presents a significant challenge when used in flexible devices, as these devices may undergo various deformations.


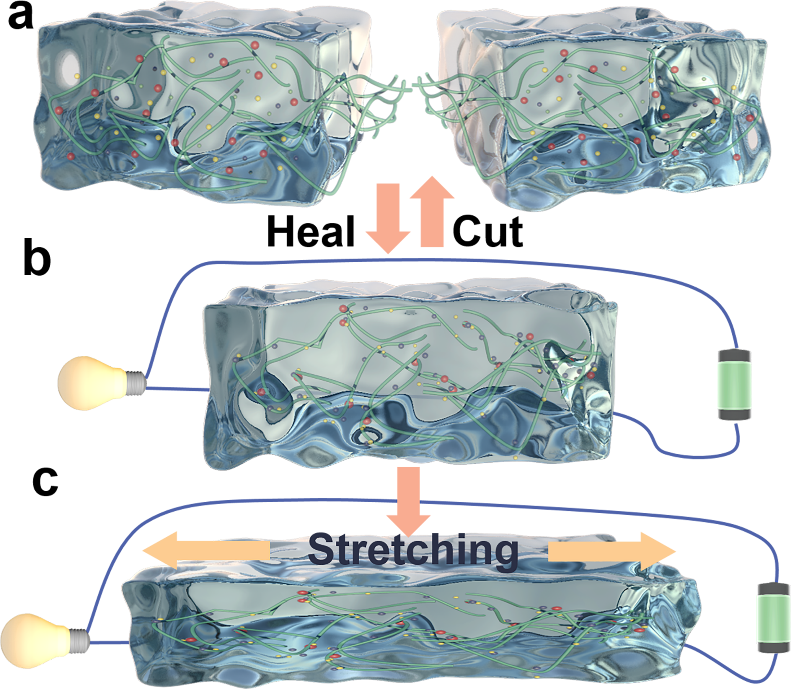


**Figure S12.** a) A diagram of the severed EG/PAAm hydrogel electrolyte b) The diagram of the conductive circuit connected by the self-healed hydrogel electrolyte c) The diagram of the self-healed hydrogel electrolyte showing its stretchability

As shown in Figure S12, the EG/PAAm hydrogel electrolyte exhibits remarkable self-healing capabilities, allowing it to remain conductive and reconnect circuits after 10 minutes of rejoining. Furthermore, the EG/PAAm hydrogel electrolyte retains outstanding tensile strength after a 4-hour self-healing process.


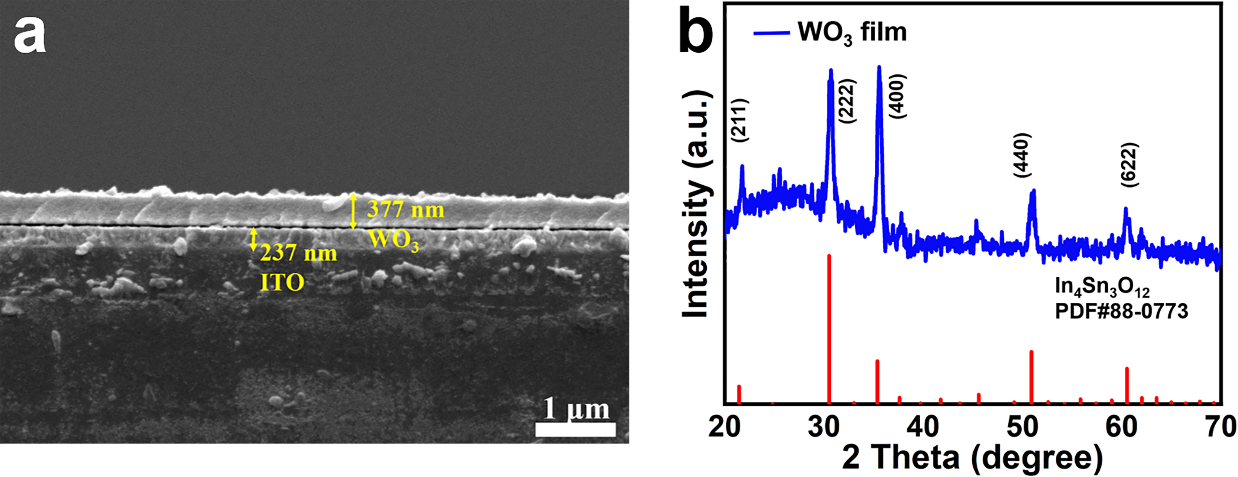


**Figure S13**. Characterization of the electrochromic WO_3_ electrode. a) The cross-sectional SEM image showcasing the thickness of the WO_3_ electrode. b) The X-ray diffraction (XRD) pattern of the WO_3_ electrode.

Figure S13a reveals that the thickness of the WO_3_ electrode is approximately 377 nm. The XRD pattern of the electrodeposited WO_3_ electrode illustrates its amorphous nature (Figure S13b).


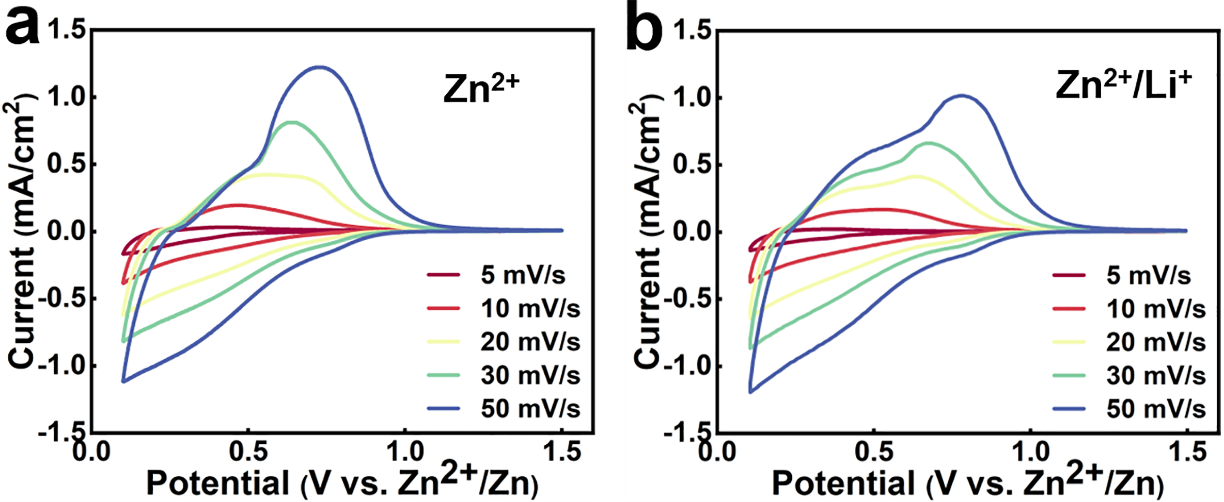


**Figure S14**. The CV curves of the WO_3_ electrode in different electrolytes. a) The WO_3_ electrode in Zn^2+^ electrolyte and b) The WO_3_ electrode in Zn^2+^/ Li^+^ electrolyte.

CV spectra of the WO_3_ electrode were measured in Zn^2+^ electrolyte and Zn^2+^/ Li^+^ electrolyte at scanning rates from 5 mV/s to 20 mV/s.


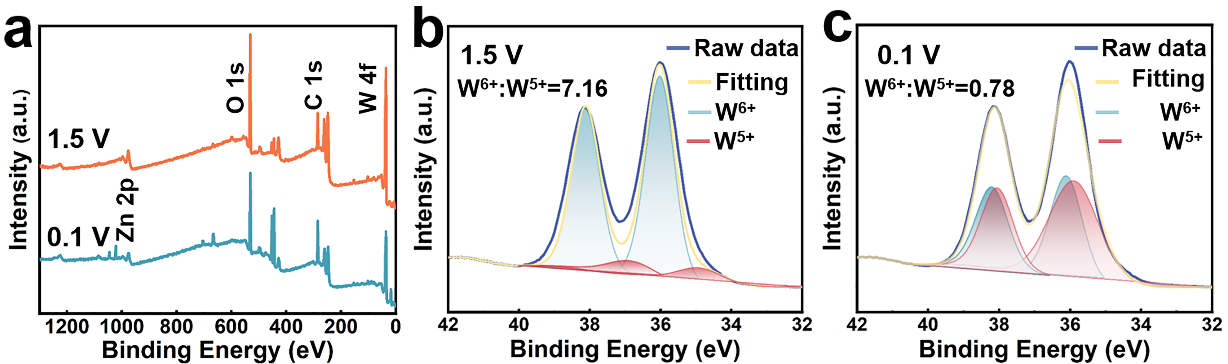


**Figure S15**. The ex-situ XPS analysis. a) Full survey spectra of the WO_3_ electrode at voltages of 1.5 V, and 0.1 V. b) W4f spectra of the WO_3_ electrode at 1.5 V. c) W4f spectra of the WO_3_ electrode at 0.1 V.

The XPS full survey spectra and high-resolution W4f XPS spectra of the WO_3_ cathode are depicted in Figure S15. The WO_3_ electrode exhibits broad multiple peaks in the W4f region, ranging from 32 to 42 eV. These multiple peaks are effectively fitted with two doublets. The first strong doublet, with peaks at 36.0 eV and 38.15 eV, is assigned to W4f7/2 and W4f5/2 of the W^6+^ species, while the second, weaker peaks at lower binding energies of 35.1 eV and 37.25 eV are attributed to the W^5+^ species. The atomic number ratios of W^6+^ and W^5+^ of the WO_3_ electrode at 1.5 V and 0.1 V are calculated to be 7.16 and 0.78, respectively. This suggests that the coloring of WO_3_ electrodes originates from the reduction of W^6+^ to W^5+^.


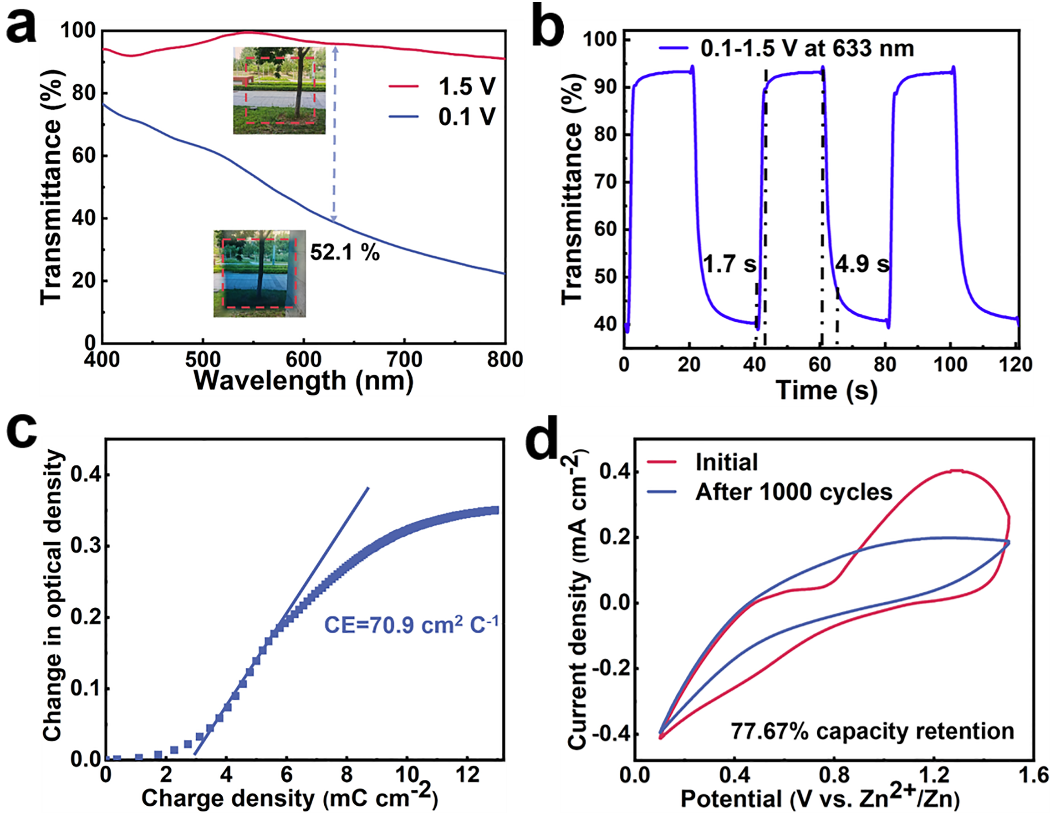


**Figure S16**. Characterization of the WO_3_ electrode in the PC-based electrolyte (i.e., 0.1 M Zn(ClO_4_)_2_ and 0.5 M LiClO_4_ in PC). a) Optical transmittance spectra of the WO_3_ electrodes at varying applied voltages (The red line and blue line represent 1.5 V and 0.1 V, respectively). Inset: corresponding digital photos of the WO_3_ electrode. b) Real-time transmittance spectrum of the WO_3_ electrode at 633 nm in the 0.1-1.5 V window. c) Changes in optical density at a light wavelength of 633 nm as a function of charge density. d) CV measurement of the WO_3_ electrode over 1000 cycles between 0.1 and 1.5 V at 100 mV/s.

As depicted in Figure S16a, the WO_3_ electrode shows an optical contrast (△T) of 52.1 % at 633 nm. The digital photographs of the WO_3_ electrode in the bleached and colored states are shown in inset. As shown in Figure S16b, the coloration time (t_c_) and bleaching time (t_b_) at 633 nm are measured to be 4.9 s and 1.7 s, respectively. Figure S16c shows that the WO_3_ electrode exhibits a coloration efficiency (CE) of 70.9 cm²C^-1^ at 633 nm in the PC-based electrolyte. Figure S16d demonstrates that the WO_3_ electrode preserves 77.67% of its initial capacity after 1000 CV cycles in the PC-based electrolyte.


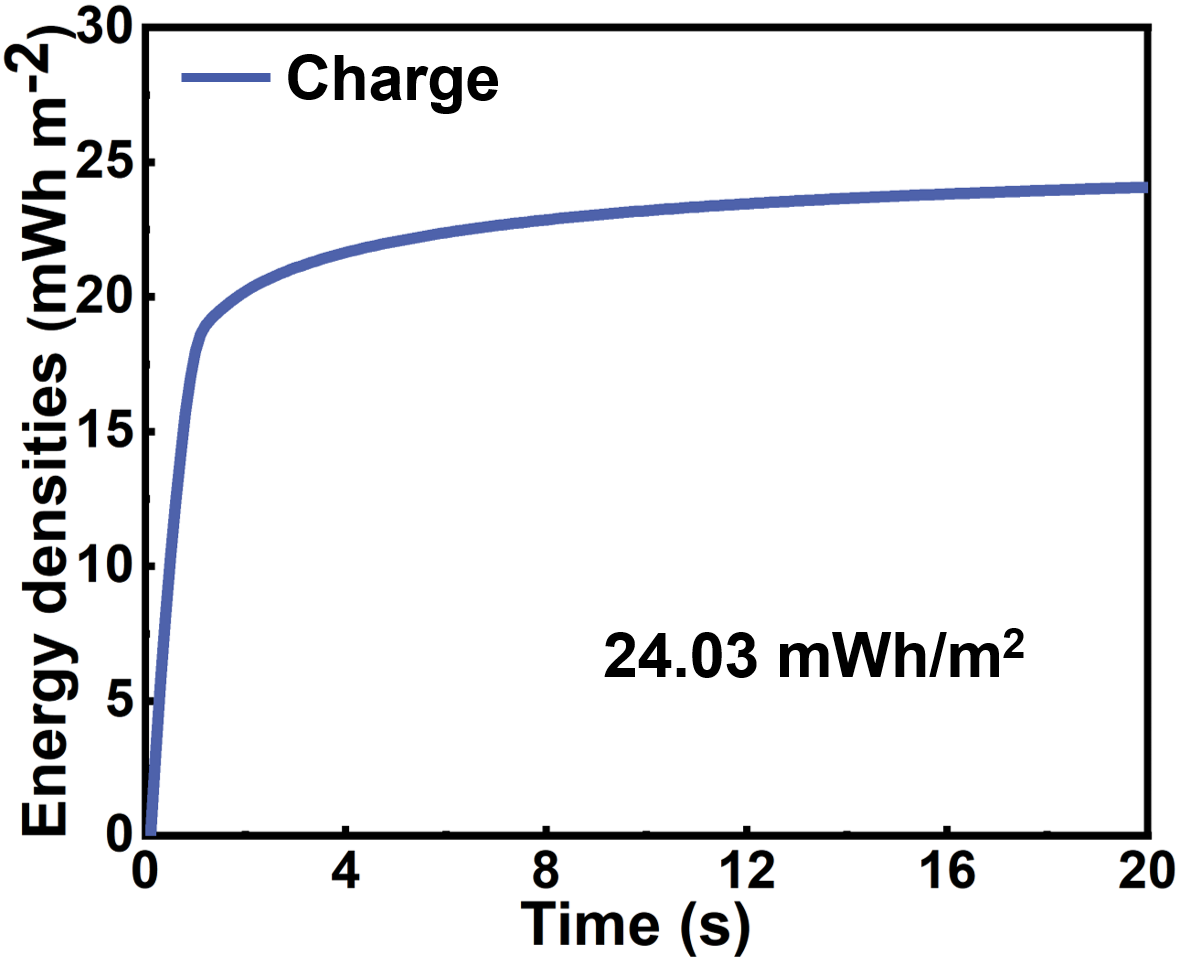


**Figure S17**. Energy density characteristics of the WO_3_ electrode measured by applying a voltage of 1.5 V for 20 s.

Figure S17 shows the input energy consumed in bleaching (i.e., charging) the WO_3_ electrode is 24.03 mWh/m^2^.


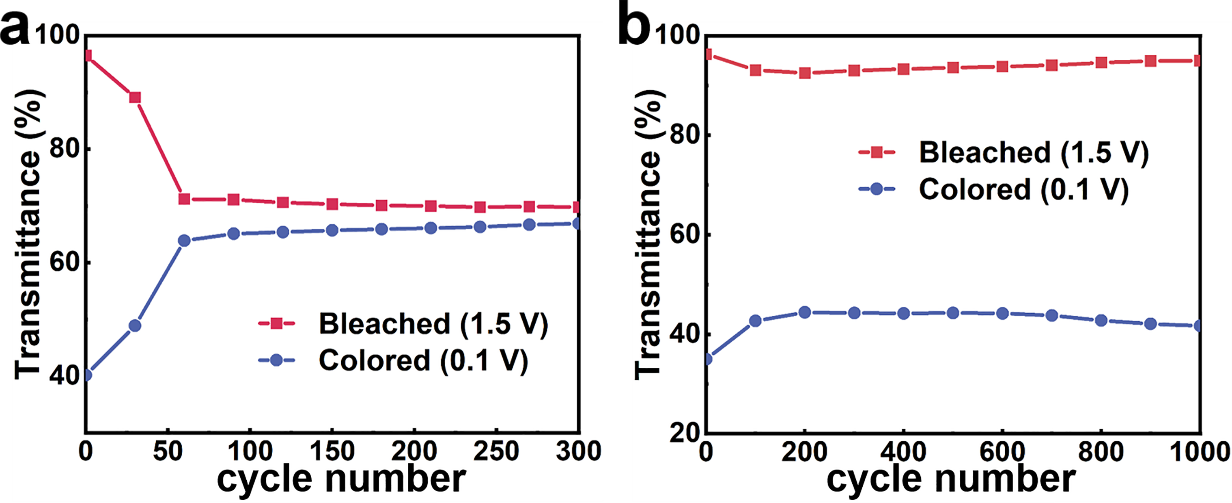


**Figure S18**. Cycling durability profiles of the WO_3_ electrode under 1.5 V and 0.1 V switching for 20 s interval in different electrolytes. a) In the aqueous electrolyte. b) In the PC-based electrolyte.

As shown in Figure S18a, the optical contrast of the WO_3_ electrode in the aqueous electrolyte decreases rapidly within the first 50 cycles, reaching only 4.2 % optical contrast after 300 cycles. In contrast, the WO_3_ electrode in the PC-based electrolyte maintains 49.2 % optical contrast after 1000 cycles, retaining 87.9 % of the initial optical contrast.


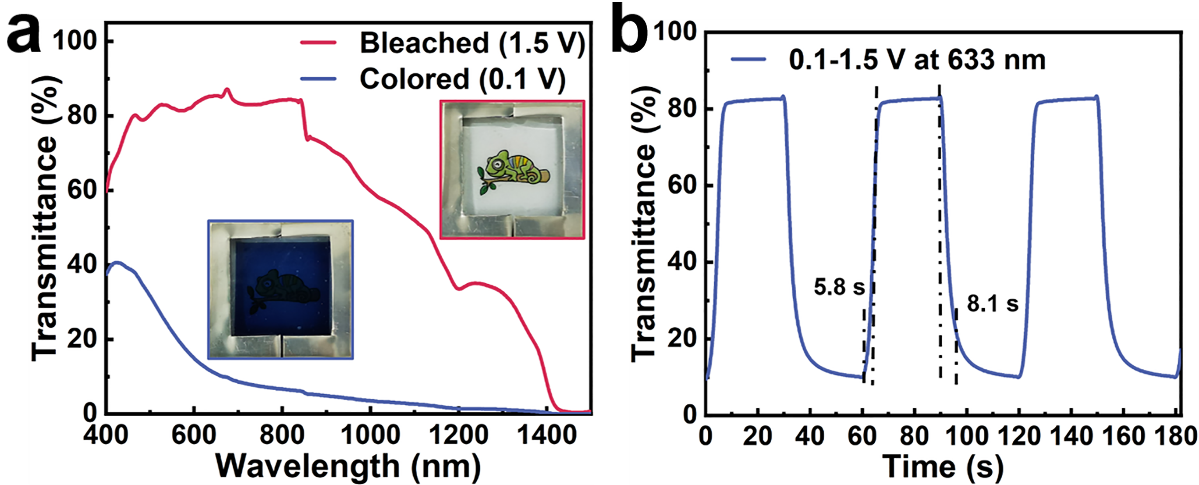


**Figure S19**. Electrochromic performance of the Zn-WO_3_ dimmer. a) Optical transmittance spectra of the Zn-WO_3_ dimmer under different voltages. Inset: corresponding digital photos of the dimmer. b) Dynamic test of the Zn-WO_3_ dimmer at 633 nm in the 0.1-1.5 V electrochemical window.

Figure S19a displays the electrochromic modulation of the Zn-WO_3_ dimmer. By applying voltages of 0.1 V and 1.5 V to the Zn-WO_3_ dimmer, a △T of 73.3% at 633 nm is achieved. Although the △T is relatively high compared to state-of-the-art electrochromic devices^[20]^, it provides limited privacy protection as the cartoon below the device remains visible to the naked eye. The dynamic transmittance characteristics of the Zn-WO_3_ dimmer, shown in Figure S19b, reveal switching times of t_c_=8.1 s and t_b_= 5.8 s.


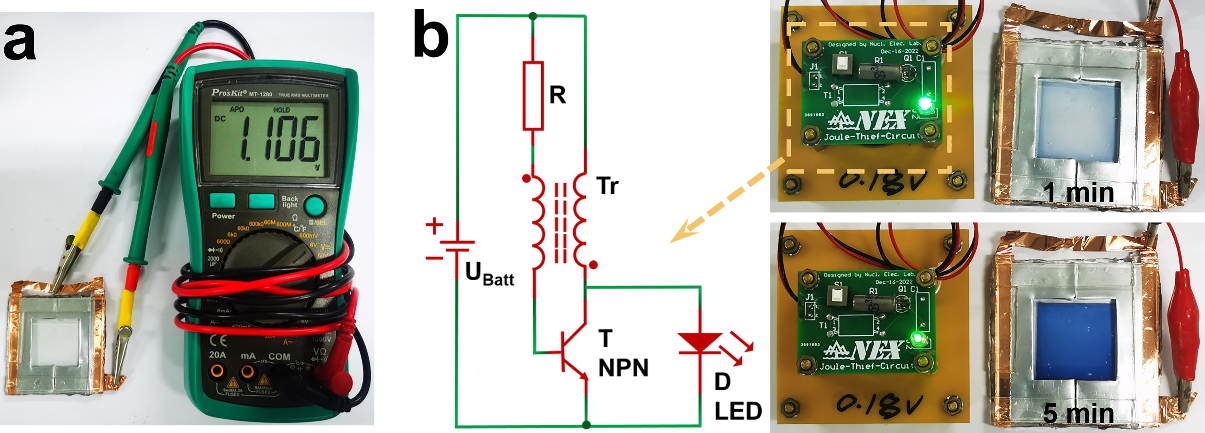


**Figure S20**. Energy retrieval capabilities of the Zn-WO_3_ dimmer. a) Digital photograph depicting the dimmer with an OCP of 1.11 V. b) Digital photograph of a 0.18 V regulated LED powered by the Zn-WO_3_ dimmer at 1 and 5 min, connected via the “Joule thief circuit”.

As shown in Figure S20a, the Zn-WO_3_ dimmer delivers an open-circuit potential (OCP) of 1.11 V. This OCP can light up an LED through the “Joule thief circuit”, alongside a spontaneous coloration process of the device (Figure S20b).


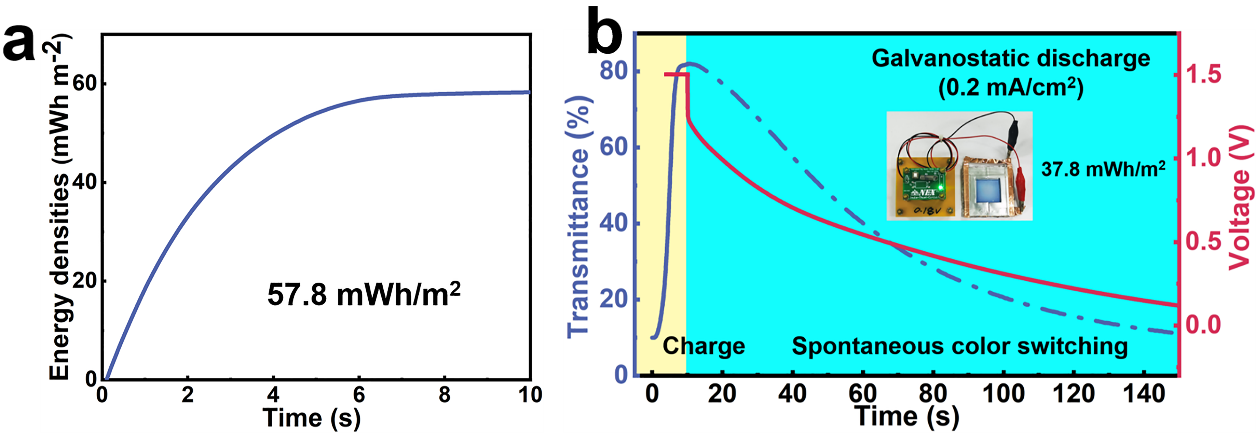


**Figure S21**. Round-trip energy efficiency of the Zn-WO_3_ dimmer. a) Energy density characteristics of the Zn-WO_3_ dimmer as determined by applying a voltage of 1.5 V for 10 s. b) Transmission at the wavelength of 633 nm during the chronoamperometric charging process at 1.5 V (solid blue line) and the galvanostatic discharge at a current density of 0.2 mA/cm^2^ (dotted blue line). The corresponding discharge curve is shown in red, with an inset showing an LED (regulated at 0.18 V) powered through the Zn-WO_3_ dimmer.

To determine the input energy density for the Zn-WO_3_ dimmer, a voltage of 1.5 V is applied for a duration of 10 seconds. The energy density profile, depicted in Figure S21a, reveals that the bleaching of the Zn-WO_3_ dimmer requires an energy input of 57.8 mWh/m². Figure S21b displays that the galvanostatic discharge process at a current density of 0.2 mA/cm² retrieves 37.8 mWh/m², along with a spontaneous coloration process. Therefore, the round-trip energy efficiency is calculated to be 65.4%.


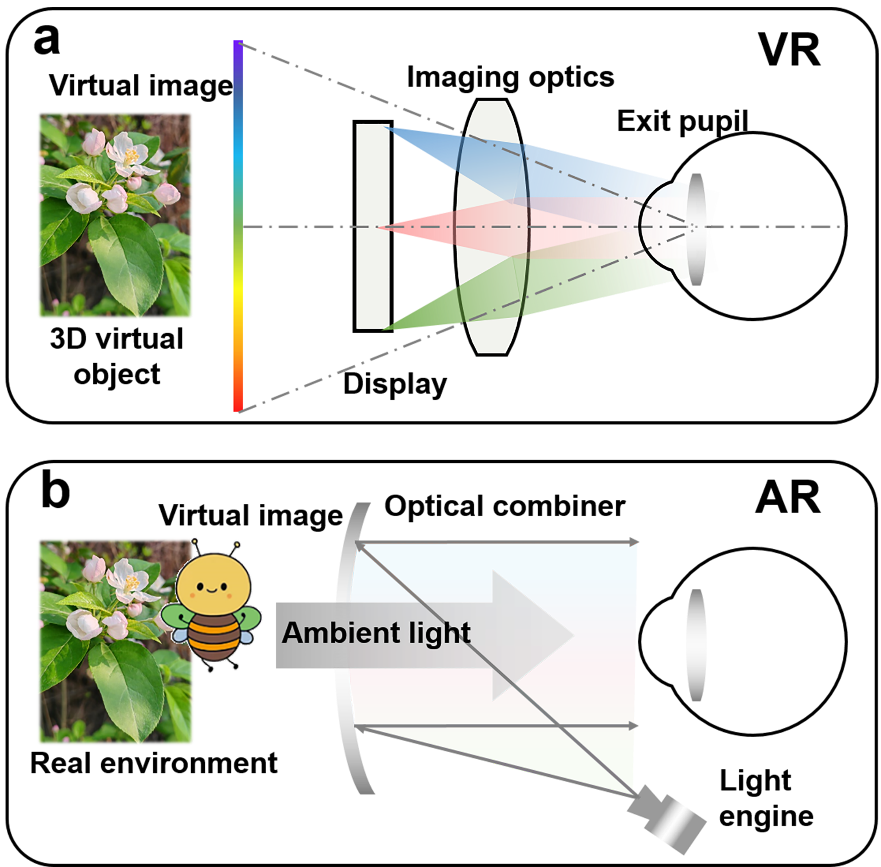


**Figure S22.** The system configurations of a) VR and b) AR system

As shown in Figure S22a, VR systems require the display of 3D virtual images on a background that is completely isolated from the external environment. The EC+PC state of the Zn-WO_3_ dimmer effectively fulfills this requirement. Moreover, the ability to overlay virtual images onto the real world is a defining feature of AR systems, as illustrated in Figure S22b. The EC state of the Zn-WO_3_ dimmer can actively modulate the incident light intensity, thereby enhancing reflected light intensity and improving image clarity.

**Table S3. Comparison of Current State-of-the-Art Photochromic Materials.**

| **Ref.** | **material** | **Photo response**  **time and**  **conditions** | **ΔT**  **（nm）** | **Bleaching**  **time and**  **conditions** | **Modulation range (nm)** |
| --- | --- | --- | --- | --- | --- |
| [21] | WO_3_ nanosheets | 5 min  UV | 45 % at 800  nm | 7 min  UV/ozone | 400-800 nm |
| [22] | WO_3_ quantum-dots | 60 s  UV | 90.8 % at 780  nm | 8 h  air/dark | 400-1500 nm |
| [23] | WO_3_ nanoparticles | 60 s  UV | ΔAbsorbance ~ 1.5 at 728  nm | 8 h  60 ^o^C | 400-800 nm |
| [24] | BaMgSiO_4_: Mn^2+^ | 60 s  310 nm illumination | ΔReflectivity 53.5 % at 524  nm | 590 nm illumination | 400-800 nm |
| [25] | Ba(Zr_0.16_Mg_0.28_Ta_0.56_)O_3_ | 3 s  365 nm illumination | 62.1 % at 505  nm | 120 s  350 ^o^C | 400-1000 nm |
| [26] | Phosphomolybdic acid | 900 s  UV | ΔAbsorbance ~ 0.9 at 760  nm | 24 h  air/dark | 400-900 nm |
| [27] | WO_3_ nanoparticles | 60 min  UV (8 W) | ΔReflectivity ~40 % | 24 h  dark | 450-2200 nm |
| [28] | Cu-doped WO_3_ nanoparticles | ~100 s  UV-light | 81 % at 1050  nm | 40 min  Room light | 400-1100 nm |
| **This work** | WO_3_ nanodots | 30 s  solar irradiance | 90.2 % at 633  nm | ~5 h  Room light | 400-1400 nm |

As shown in Table S3, the photochromic process generally requires longer bleaching times compared to the electrochromic process, mainly due to the slow self-oxidation of the photochromic active material. Future advancements are expected to introduce new methods that could significantly reduce the bleaching time of photochromic materials.

**Supplementary References**

[1] Y. Ma, Y. Wang, J. Zhou, Y. Lan, S. Jiang, Y. Ge, S. Tan, S. Zhang, C. Wang, Y. Wu, Mater. Horiz., **2024**, *11*, 3825.

[2] H. Ling, J. Wu, F. Su, Y. Tian, Y. Liu, Nat. Commun., **2021**, *12*, 1010.

[3] B. Deng, Y. Zhu, X. Wang, J. Zhu, M. Liu, M. Liu, Y. He, C. Zhu, C. Zhang, H. Meng, *Adv. Mater.* **2023**, *35*, 2302685.

[4] Z. Zhou, Y. Tang, F. Zhao, G. Li, G. Xu, Y. Liu, G. Han, Chem. Eng. J., **2024**, *481*. 148724.

[5] P. Sun, J. Chen, Y. Li, X. Tang, H. Sun, G. Song, X. Mu, S. Cong, Z. Zhao, InfoMat., **2023**, *2*, 12363.

[6] K. Wang, H. Wang, J. Li, Y. Liang, X. Xie, J. Liu, C. Gu, Y. Zhang, G. Zhang, C. Liu, Mater. Horiz., **2021**, *8*, 2520.

[7] Le. Wang, X. Zhang, X. Chen, X. Li, Y. Zhao, W. Li, J. Zhao, Z. Chen, Y. Li, J. Mater. Chem. C, **2021**, *9*, 1641.

[8] H. Li, C. J. Firby, A. Y. Elezzabi, *Joule* **2019**, *3*, 2268.

[9] F. Zhao, C. Li, S. Li, B. Wang, B. Huang, K. Hu, L. Liu, W. W. Yu, H. Li, *Adv. Mater.* **2024**, *36,* 2405035.

[10] S. Zhang, Y. Peng, J. Zhao, Z. Fan, B. Ding, J. Y. Lee, X. Zhang, Y. Xuan, *Adv. Optical Mater.* **2022**, *11*, 2202115.

[11] S. Zhang, S. Cao, T. Zhang, J. Y. Lee, *Adv. Mater.* **2020**, *32*, 2004686.

[12] W. Zhang, H. Li, M. Al-Hussein, A. Y. Elezzabi, *Adv. Opt. Mater.* **2019**, *8*, 1901224.

[13] J. Li, J. Li, H. Li, C. Wang, M. Sheng, L. Zhang, S. Fu, *ACS Appl Mater Interfaces* **2021**, *13*, 27200.

[14] R. Ren, S. Liu, Y. Gao, P. Lei, J. Wang, X. Tong, P. Zhang, Z. Wang, G. Cai, *ACS Energy Lett.* **2023**, *8*, 2300.

[15] D. Ma, H. Niu, J. Huang, Q. Li, J. Sun, H. Cai, Z. Zhou, J. Wang, *Nano Lett.* **2024**, *24*, 814.

[16] P. Liu, B. Wang, C. Wang, L. Ma, W. Zhang, E. Hopmann, L. Liu, A. Y. Elezzabi, H. Li, *Adv. Funct. Mater.* **2024**, 2400760.

[17] Y. Sang, Y. Ma, G. Li, K. Cui, M. Yang, H. Chen, Y. Li, *Chem. Eng. J.* **2023**, *463*,142256

[18] a)P. Zou, X. Hong, Y. Ding, Z. Zhang, X. Chu, T. Shaymurat, C. Shao, Y. Liu, J. Phys. Chem. C **2012**, *9*, 5787;b)J. Wei, X. Jiao, T. Wang, D. Chen, ACS Appl. Mater. Interfaces **2016**, *8*, 29713

[19] a)X. Zhu, F. Zhang, L. Zhang, L. Y. Zhang, Y. Z. Song, T. Jiang, S. Sayed, C. Lu, X. G. Wang, J. Y. Sun, Z. F. Liu, Adv. Funct. Mater. **2018**, *28*, 1705015; b)C. Xu, Y. Sun, J. Zhang, W. Xu, H. Tian, Adv. Energy Mater. **2022**, *12*, 2201542; c)F. Zhao, J. Zhao, Y. Zhang, X. Wang, W. Wang, J. Mater. Chem. C, **2021**, *9*, 7958

[20] a)W. Zhang, H. Li, A. Y. Elezzabi, *Adv. Funct. Mater.* **2023**, 2300155; b)S. Z. Sheng, J. L. Wang, B. Zhao, Z. He, X. F. Feng, Q.G. Shang, C. Chen, G. Pei, J. Zhou, J.W. Liu, S.H. Yu, *Nat. Commun.* **2023**, *14*, 3231.

[21] J. Wei, X. Jiao, T. Wang, D. Chen, J. Mater. Chem.C **2015**, 3, 7597.

[22] Y. Zhu, Y. Yao, Z. Chen, Z. Zhang, P. Zhang, Z. Cheng, Y. Gao, Sol. Energy Mater. Sol. Cells **2022**, 239.

[23] J. Yang, C. Tang, H. Sun, Z. Liu, Z. Liu, K. Li, L. Zhu, G. Qin, G. Sun, Y. Li, Q. Chen, ACS Appl. Mater. Interfaces **2021**, 13, 31180.

[24] Z. Yang, J. Du, L. I. D. J. Martin, A. Feng, E. Cosaert, B. Zhao, W. Liu, R. Van Deun, H. Vrielinck, D. Poelman, Adv. Opt. Mater. **2021**, 9, 2100669.

[25] W. Tang, C. Zuo, C. Ma, Y. Wang, Y. Li, X. Yuan, E. Wang, Z. Wen, Y. Cao, Chem. Eng. J. **2022**, 435, 134670.

[26] T. Yimyai, D. Crespy, A. Pena-Francesch, Adv. Funct. Mater. **2023**, 33, 2213717.

[27] Y. Badour, M. Pedros, M. Gaudon, S. Danto, Adv. Opt. Mater. **2024**, 12, 2301717.

[28] W. Meng, A. J. J. Kragt, Y. Gao, E. Brembilla, X. Hu, J. S. van der Burgt, A. P. H. J. Schenning, T. Klein, G. Zhou, E. R. van den Ham, L. Tan, L. Li, J. Wang, L. Jiang, Adv. Mater. **2023**, 36, 2304910.
